# Mass spectrometric analysis of purine *de novo* biosynthesis intermediates

**DOI:** 10.1371/journal.pone.0208947

**Published:** 2018-12-10

**Authors:** Lucie Mádrová, Matyáš Krijt, Veronika Barešová, Jan Václavík, David Friedecký, Dana Dobešová, Olga Součková, Václava Škopová, Tomáš Adam, Marie Zikánová

**Affiliations:** 1 Institute of Molecular and Translational Medicine, Faculty of Medicine and Dentistry, Palacký University and University Hospital in Olomouc, Olomouc, Czech Republic; 2 Department of Clinical Biochemistry, University Hospital in Olomouc, Olomouc, Czech Republic; 3 Research Unit for Rare Diseases, Department of Pediatrics and Adolescent Medicine, First Faculty of Medicine, Charles University and General University Hospital in Prague, Prague, Czech Republic; 4 Laboratory of Inherited Metabolic Disorders, Department of Clinical Chemistry, University Hospital in Olomouc, Olomouc, Czech Republic; University of Florida, UNITED STATES

## Abstract

Purines are essential molecules for all forms of life. In addition to constituting a backbone of DNA and RNA, purines play roles in many metabolic pathways, such as energy utilization, regulation of enzyme activity, and cell signaling. The supply of purines is provided by two pathways: the salvage pathway and *de novo* synthesis. Although purine *de novo* synthesis (PDNS) activity varies during the cell cycle, this pathway represents an important source of purines, especially for rapidly dividing cells. A method for the detailed study of PDNS is lacking for analytical reasons (sensitivity) and because of the commercial unavailability of the compounds. The aim was to fully describe the mass spectrometric fragmentation behavior of newly synthesized PDNS-related metabolites and develop an analytical method. Except for four initial ribotide PDNS intermediates that preferentially lost water or phosphate or cleaved the forming base of the purine ring, all the other metabolites studied cleaved the glycosidic bond in the first fragmentation stage. Fragmentation was possible in the third to sixth stages. A liquid chromatography-high-resolution mass spectrometric method was developed and applied in the analysis of CRISPR-Cas9 genome-edited HeLa cells deficient in the individual enzymatic steps of PDNS and the salvage pathway. The identities of the newly synthesized intermediates of PDNS were confirmed by comparing the fragmentation patterns of the synthesized metabolites with those produced by cells (formed under pathological conditions of known and theoretically possible defects of PDNS). The use of stable isotope incorporation allowed the confirmation of fragmentation mechanisms and provided data for future fluxomic experiments. This method may find uses in the diagnosis of PDNS disorders, the investigation of purinosome formation, cancer research, enzyme inhibition studies, and other applications.

## Introduction

**Fig 1:**
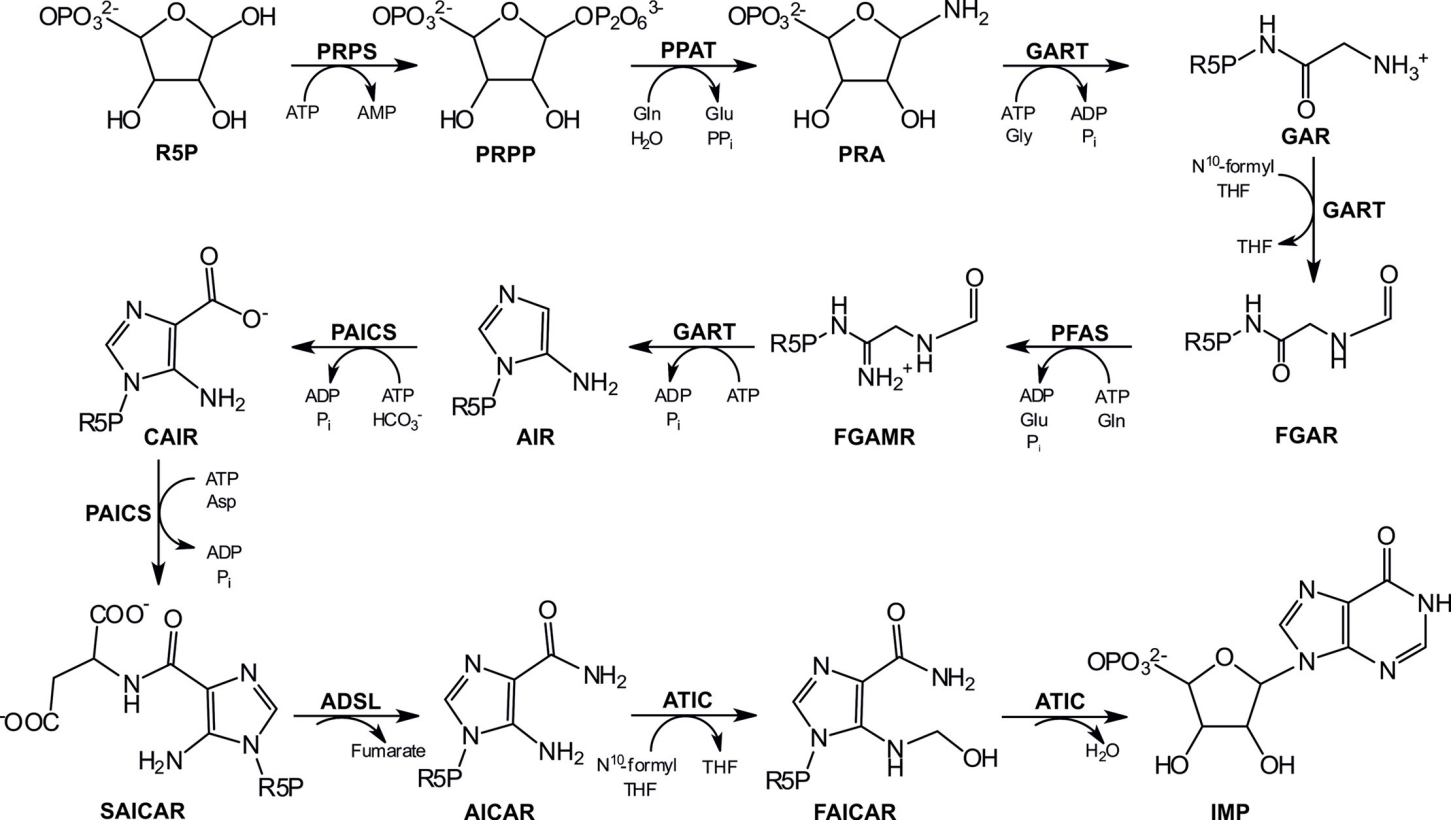
Purine *de novo* synthesis in humans. Abbreviations: phosphoribosylamine (PRA), glycinamideribonucleotide (GAR), N-formylglycinamide ribonucleotide (FGAR), N-formylglycinamidine ribonucleotide (FGAMR), aminoimidazole ribonucleotide (AIR), carboxyaminoimidazole ribonucleotide (CAIR), N-succinocarboxamide-5-aminoimidazole ribonucleotide (SAICAR), 5-aminoimidazole-4-carboxamide ribonucleotide (AICAR), 5-formamido-4-imidazolecarboxamide ribonucleotide (FAICAR), inosine-5-monophosphate (IMP), glutamine (Gln), glutamate (Glu), pyrophosphate (PPi), adenosine-5'-triphosphate (ATP), adenosine 5'-diphosphate (ADP), glycine (Gly), phosphate (Pi), N^10^-formyl tetrahydrofolate (N^10^-formyl THF), tetrahydrofolate (THF), hydrogen carbonic acid (HCO^3-^), aspartate (Asp). For enzyme abbreviations, see the second paragraph of the Introduction.

Purine nucleotides have vital functions in numerous pathways in both prokaryotes and eukaryotes. As a part of many essential biomolecules, purine nucleotides participate in nucleic acid synthesis, transcription, translation, cell signaling processes, and maintaining energetic homeostasis and act as cofactors, neuromodulators, and cotransmitters. The supply of purine nucleotides is provided by two pathways: the salvage pathway and *de novo* synthesis. Purine *de novo* synthesis (PDNS) is a sequence of ten reactions catalyzed by six enzymes. Three of these enzymes are multifunctional in PDNS (Fig 1), and bifunctional adenylosuccinate lyase (ADSL) also participates in the purine nucleotide cycle, catalyzing the conversion of adenylosuccinic acid (SAMP) to adenosine monophosphate (AMP).

Initially, ribose-5-phosphate (R5P), which is synthesized in the pentose phosphate pathway, is converted by ribose-phosphate pyrophosphokinase (PRPS, EC 2.7.6.1) to PRPP, the first metabolite of PDNS. The formation of PRA is catalyzed by amidophosphoribosyltransferase (PPAT, EC 2.4.2.14). The trifunctional enzyme GART (phosphoribosylglycinamide synthetase, EC 6.3.4.13; phosphoribosylglycinamide formyltransferase, EC 2.1.2.2; and phosphoribosylaminoimidazole synthetase, EC 6.3.3.1) catalyzes steps 2, 3, and 5, respectively. Formation of FGAMR from FGAR is catalyzed by phosphoribosylformylglycinamidine synthase (PFAS, EC 6.3.5.3). The bifunctional enzyme PAICS consists of phosphoribosylaminoimidazole carboxylase (EC 4.1.1.21, step 6) and phosphoribosylaminoimidazolesuccinocarboxamide synthase (EC 6.3.2.6, step 7). The enzyme ADSL (EC 4.3.2.2) catalyzes the formation of AICAR from SAICAR. The last two steps are catalyzed by the bifunctional ATIC enzyme phosphoribosylaminoimidazolecarboxamide formyltransferase (EC 2.1.2.3, step 9) and IMP cyclohydrolase (EC 3.5.4.10, step 10). PDNS contributes to the cellular pool of purines at times when there is an increased demand for purines, particularly during the G1 and S phases of the cell cycle, and supplies enough purines for the synthesis of RNA and DNA [1]. The highest activity of PDNS has been found in the skeletal muscles, even exceeding the activity in the liver. In contrast, minimal activity has been found in the heart and brain (except developing brains). Erythrocytes, as an example of nondividing cells, lack an intact purine *de novo* synthetic pathway [2].

Recent studies focusing on protein-protein interactions have revealed that the individual enzymes of PDNS group together sequentially to form multienzyme complexes called purinosomes, which facilitate the synthesis of purines. It has been proposed that the formation of purinosomes inside cells occurs in the G1 and S phases of the cell cycle [1]. This is well described by immunofluorescence imaging of mammalian cells under purine-rich or purine-depleted conditions [1, 3, 4] and by monitoring the overall flux through the pathway [4]. Direct tracing of the intermediates of the pathway has thus far only been possible with the use of radioactive labeling [5].

To date, two genetically determined defects of this metabolic pathway (out of ten that are theoretically possible) have been identified: ADSL deficiency (OMIM 103050) and AICA-ribosiduria (ATIC deficiency, OMIM 608688). Almost eighty cases of ADSL deficiency [6] and only one case of AICA-ribosiduria [7] have been reported worldwide to date. There are three possible explanations for this limited number of cases: the first two are that the clinical conditions associated with these particular enzyme deficiencies are either benign or incompatible with fetal development. The third reason is that the available screening/diagnostic tools are relatively laborious and expensive, which limits their widespread use. The methods used for studying purine metabolism and diagnosing related metabolic diseases include proton nuclear magnetic resonance, electrophoresis, and chromatography. Chromatography dominates the field and has historically used UV absorbance, radiometric and tandem mass spectrometric detection [4, 8–10]. PDNS ribosides excreted into growth media in four genetically modified cell lines were detected in a recent study [11]. Currently, there is no published method for the detection of all natural PDNS intermediates. The only published report utilizes radioactive tracing [5]. Mass spectrometric fragmentation analysis of the compounds is of value because of the biological importance of such compounds and the lack of experimental fragmentation spectra. Mass spectral databases contain either no PDNS metabolites or *in silico* fragmentation spectra of the metabolites, as these compounds are not commercially available (except AICAR, AICAr and SAMP).

The aim of this work is to fully describe the mass spectrometric fragmentation behavior of PDNS metabolites. Based on this knowledge, we analyzed individual PDNS-defective cell lines to describe metabolic changes in the pathway with the objective of predicting metabolic changes in analogous human conditions. The method that was developed for the detection of PDNS intermediates could serve in pathway kinetic studies (enzyme activity measurements or inhibition experiments, since a number of compounds are claimed to be PDNS inhibitors) and in the diagnostics of inherited metabolic disorders. The method can also be applied in cancer research, as some genes encoding PDNS enzymes have been reported to be up- or downregulated in some tumors [12, 13]. In the first part, we synthesized 16 metabolites of PDNS and their dephosphorylated analogues and performed in-depth multistage mass spectrometric fragmentation analysis.

In the second part, we analyzed edited HeLa cells that were enzymatically deficient in the individual steps of PDNS and treated with isotopically labeled glycine. The aim was to confirm the identity of the newly synthesized intermediates of PDNS by comparing the fragmentation patterns of the synthesized intermediates with those produced by cells (formed under pathological conditions of known and theoretical possible defects of PDNS). Data obtained from the comparison of high-resolution mass spectrometry (HRMS) fragmentation and mass shifts in labeled and natural intermediates provided additional structural certainty in the form of knowledge of the labeling position.

Finally, experiments with PDNS-deficient HeLa cell lines provided data for stable isotope fluxomic analysis and allowed a deeper understanding to be gained of the biochemical changes under those circumstances.

## Materials and methods

### Chemicals

Isotopically labeled glycine (U-^13^C_2_, ^15^N) was purchased from Cambridge Isotope Laboratories (Andover, MA, USA). AICAR, 5-aminoimidazole-4-carboxamide riboside (AICAr) and adenylosuccinic acid (SAMP) were purchased from Toronto Research Chemicals Inc. (North York, Canada). SAICAR, succinylaminoimidazolecarboxamide riboside (SAICAr), succinyladenosine (SAdo), AIR, 5-aminoimidazole riboside (AIr), CAIR, carboxyaminoimidazole riboside (CAIr), and N^10^-formyl-tetrahydrofolate (N^10^-formyl-THF) were prepared as previously described [11, 14]. Calf intestinal alkaline phosphatase (CIP) and NEB3 buffer were purchased from New England Biolabs (NEB, Ipswich, MA, USA), and Dulbecco's minimum essential medium (DMEM), F12 nutrient mix, and fetal bovine serum (FBS) were obtained from Life Technologies, ThermoFisher Scientific (MA, USA). Minimum Essential Medium (MEM) was obtained from BioSera (Nuaille, France). All other chemicals were purchased from Sigma-Aldrich (St. Louis, USA).

### Preparation and purification of substrates

GAR, FGAR, and FGAMR intermediates were synthesized biochemically using bacterial recombinant enzymes. FAICAR was prepared by inorganic synthesis. We did not attempt to synthesize PRA, as it is known to be unstable *in vivo* [15, 16].

The initial concentration of all compounds ranged from 57 μM in samples of FGAMR/r to 124 μM in samples of GAR/r (see S1 Table).

The bacterial recombinant enzymes phosphoribosylglycinamide synthetase (GARS) and phosphoribosylglycinamide formyltransferase (GARTF) were expressed and purified as fused protein maltose binding protein MBP-GARS and MBP-GARTF using the pMAL^TM^ Protein Fusion and Purification System (New England Biolabs Inc., USA), as described previously [17].

To produce recombinant bacterial phosphoribosylformylglycinamide synthetase fused with a C-terminal polyhistidine tag (6H-PurL), the gene was introduced into the p6H vector, expressed in *Escherichia coli*, and purified on a Co^2+^-immobilized metal affinity chromatography column (GE Healthcare) according to standard procedure.

#### GAR/r preparation

The reaction mixture containing 5.7 mM ribose-5-phosphate, 0.7 mM ATP, 10 mM glycine, 10 mM ammonium hydroxide, 12.7 mM magnesium chloride, 20 mM phosphate buffer pH 7.4, and 0.4 μg/μL purified MBP-GARS was incubated at 37°C for four hours. The reaction was analyzed by high-performance liquid chromatography coupled with mass spectrometry (HPLC-MS). The riboside form was prepared by dephosphorylation with 1 U of CIP from NEB at 37°C for four hours.

#### FGAR/r preparation

The reaction mixture containing 5.7 mM ribose-5-phosphate, 0.7 mM ATP, 10 mM glycine, 10 mM ammonium hydroxide, 12.7 mM magnesium chloride, 0.1 mM N^10^-formyl-THF, 20 mM phosphate buffer pH 7.4, 0.4 μg/μL MBP-GARS, and 0.4 μg/μL MBP-GARTF was incubated at 37°C for four hours. The subsequent procedure was the same as in GAR/r preparation.

#### FGAMR/r preparation

A total of 200 μL of the reaction mixture from the synthesis of FGAR was incubated with 2 mM glutamine, 2 mM ATP, and 0.25 μg/μL of purified 6H-PurL at 37°C for four hours. The subsequent procedure was the same as in GAR/r preparation.

#### FAICAR/r preparation

FAICAr was prepared according to Lukens et al. [18]. FAICAR was prepared by adjusting the procedure used for the synthesis of FAICAr. In brief, we incubated 10 mg of AICAR with 11 mg of NaOH, 136 μL of formic acid and 250 μL of acetic anhydride for 1 hour at 37°C. The product of the reaction was analyzed by HPLC-MS.

### Cell cultivation and harvesting

We used the CRISPR-Cas9 genome-edited HeLa cells CR-GART, CR-PFAS, CR-PAICS, CR-ADSL, and CR-ATIC prepared by Baresova et al. in 2016 [11]. CR-HGPRT cells (hypoxanthine-guanine phosphoribosyltransferase deficient) were prepared analogously [19]. HeLa cells were cultured in a humidified atmosphere and incubated with 5% CO_2_ at 37°C. All cells (knockout and control) were maintained in DMEM/F12 nutrient mix medium supplemented with 10% FBS (Gibco, Invitrogen) and 1% penicillin/streptomycin. The medium of the knockout cells was enriched with 3x10^-5^ M of adenine. Twenty-four hours prior to the experiment, all the cell types were cultivated in purine-depleted DMEM supplemented with dialyzed 10% FBS [11] and 1% penicillin/streptomycin. Two hours prior to cell harvesting, the cells were washed with PBS and placed into 5 mL of glycine-free MEM with 500 μM of isotopically labeled glycine (U-^13^C_2_, ^15^N) added. Each deficient cell line was cultivated in hexaplicate in 75-cm^2^ flasks (approx. 5 million cells).

Cells were harvested by means of the modified quenching method described by Wojtowicz et al. [20]. Initially, the medium was transferred into a 15-mL plastic tube for subsequent analytical preparation. Cellular metabolism was quenched by spraying 40 mL of 60% aqueous cold methanol (v/v, -50°C) by means of a plastic syringe with a needle. The culture flasks were kept on ice and extracted with 1 mL of 80% methanol (v/v, -50°C), and the cells were mechanically detached using a cell scraper. The cell debris was drained out with a pipette. For an additional extraction, another 2 mL of cold methanol was added. The methanol extracts were combined, sonicated (30 s), and centrifuged (1800 *g*, 5 min, 4°C), and the supernatants were freeze-dried.

### Preparation of cell lysates

A total of 500 μL of cold 80% methanol was added to each lyophilizate and thoroughly mixed. The samples were centrifuged at 15,000 *g* for 15 min at 4°C, and the supernatants were taken for analysis.

### Preparation of cell media

The media were mixed using a vortex; then, 100 μL of each sample was taken and 300 μL of 80% methanol was added. The samples were left at -80°C overnight. The extracts were centrifuged at 15,000 *g* for 15 min at 4°C, and the supernatants were analyzed.

### Fragmentation analysis of PDNS intermediates and their dephosphorylated analogues (HPLC-HRMS^n^)

Chromatographic separation was achieved with hydrophilic interaction liquid chromatography using an Ultimate 3000 RS (ThermoFisher Scientific, MA, USA). The aminopropyl column (Luna NH_2_ 3 μm 100 Å, 100 x 2 mm, Phenomenex, Torrance, USA) was maintained at 35°C. The mobile phase consisted of 20 mM ammonium acetate in water at pH 9.75 (mobile phase A) and acetonitrile (mobile phase B). The gradient elution was performed as follows: t = 0.0, 95% B; t = 7.0–13.0, 10% B; t = 14.0–17.0, 95% B. The flow rate was set to 0.3 mL/min, and the injection volume was 2 μL.

Multistage fragmentation analysis was performed on an Orbitrap Elite (ThermoFisher Scientific, MA, USA) operating in positive mode using electrospray ionization (capillary temperature 350°C, source heater temperature 300°C, sheath gas 10 arb. units, auxiliary gas 35 arb. units, sweep gas 0 arb. units). The electrospray voltage was set at +3.0 kV. Fragmentation for the most abundant fragments with intensities higher than 1E4 was performed using data-dependent analysis (DDA) or TreeRobot (HighChem, SK); otherwise, the selection of fragments was performed manually. Up to five of the most intense signals in MS^2^ were isolated and further fragmented. Then, one to six of the most intense signals from each MS^3^ spectrum were subjected to fragmentation to MS^4^. The subsequent MS^n^ stages were also dependent on the intensities of the emerging fragments, usually producing spectra of the one or two most intense fragments from MS^4^/MS^5^. The fragmentation spectra were produced via collision-induced dissociation (CID) using 30 units of normalized collision energy; the isolation width was 2 Da, and the injection time was 200 ms. All the fragmentation spectra were measured with a resolution of 120,000 full-width at half-maximum (FWHM) and with a mass error below 3 ppm. The multistage fragmentation spectra of each compound were organized into mass spectral trees. In every spectrum, the structures of the fragments belonging to the precursor (target) compound/fragment were identified with the predictive fragmentation software MassFrontier 7.0.5.09 SP3 (HighChem, SK).

### Analysis of cell lysates and media

The chromatographic conditions were the same as in the HPLC-HRMS^n^ analysis mentioned above. Detection was performed on an Orbitrap Elite operating in positive ionization mode with the same setting as above. The detection method was divided into four time segments. Full scan analysis within the mass range *m/z* 70–1000 was performed in the first (0.0–3.0 min) and fourth (12.0–17.0 min) segments. The selected ion monitoring (SIM) method was applied in the second segment (3.0–7.0 min) for the analysis of ribosides (*m/z* 177–417) and in the third segment (7.0–12.0 min) for the analysis of ribotides (*m/z* 257–497) to enhance the sensitivity towards these metabolites (except for the measurement of SAdo and SAMP, which had different *m/z* ranges: 379–389 and 459–469, respectively). The resolution was set to 60,000 FWHM. The mass error was below 3 ppm. All cell lines were measured in hexaplicate, and the intensity values are presented as averages. The identities of the accumulated compounds in both cell lysates and media were confirmed by MS^2^ fragmentation analysis. Fragmentation spectra were produced via CID with the fragmentation energy set to 30 units of normalized collision energy.

## Results and discussion

### Preparation and purification of PDNS intermediates and their dephosphorylated analogues

The ribosides AIr, CAIr, SAICAr, AICAr, FAICAr, and SAdo and ribotides AIR, CAIR, and SAICAR were synthesized according to previously published procedures [11, 14]. AICAR and SAMP were commercially available.

New methods have been developed for the preparation of GAR, FGAR, and FGAMR. The strategies were based on the preparation of the bacterial recombinant enzymes MBP-GARS, MBP-GARTF, and 6H-PurL. The GAR metabolite was produced in two reactions that occurred in one step from ribose-5-phosphate. The first reaction was the formation of PRA from ribose-5-phosphate and ammonium hydroxide as a function of pH as described earlier [11]. In the second reaction, PRA was converted by MBP-GARS to GAR with a production yield of 10%. Then, GAR was dephosphorylated to GAr via CIP. FGAR was produced in the same reaction as GAR with added N^10^-formyl THF and MBP-GARTF. The resulting FGAR was dephosphorylated to FGAr by CIP. FGAMR was prepared from FGAR in the reaction catalyzed by the 6H-PurL enzyme and dephosphorylated by CIP to FGAMr.

FAICAR/FAICAr was prepared inorganically from AICAR/AICAr. We applied a formylating environment consisting of formic acid, acetic anhydride, and NaOH to convert AICAR to FAICAR within one hour at 37°C.

### MS^n^ fragmentation analysis of PDNS intermediates and their dephosphorylated analogues

In total, eight biologically stable PDNS intermediates (GAR, FGAR, FGAMR, AIR, CAIR, AICAR, SAICAR, and FAICAR) and their dephosphorylated forms (GAr, FGAr, FGAMr, AIr, CAIr, AICAr, SAICAr, and FAICAr) were subjected to HRMS^n^ fragmentation analysis. Moreover, other PDNS-related purine metabolites, SAMP, SAdo, and IMP, were measured.

**Fig 2:**
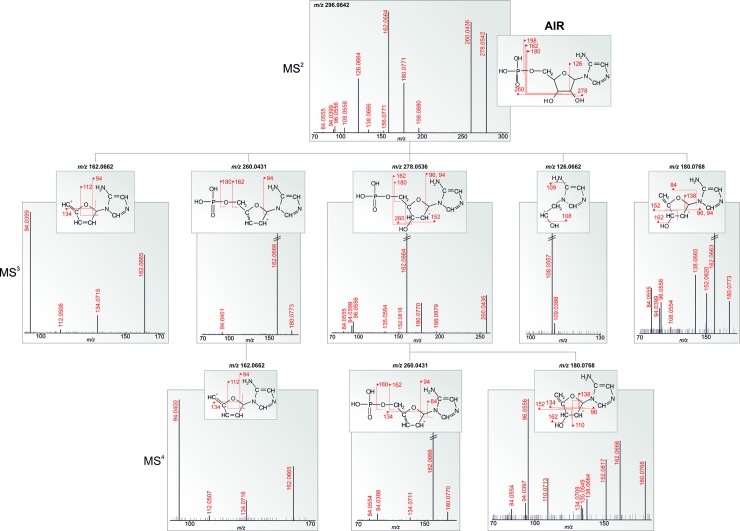
Fragmentation spectral tree of AIR. Every *m/z* assigned with accurate mass in the spectra (in red) belongs to the fragmented structure. The other *m/z* represent coeluting compounds or masses belonging to the fragmented structure that could not be identified using the given procedure.

The compounds were sequentially fragmented up to MS^6^. The structure of the fragmented compound or fragment is shown in every spectrum, and up to six of the most intense fragments are depicted in the structure (see one example in Fig 2; the rest are provided in S1 Fig). More than half of the compounds were fragmented up to MS^4^. The number of fragmentation steps was determined by the concentration of the synthesized compound in the reaction mixture (57–124 μM), molecular mass, ionization efficiency in positive mode and ion suppression (relatively minor effect due to experimental set-up). In our view, the richest information about the fragmentation behavior of PDNS metabolites lies within the MS^3^ and MS^4^ spectra. Higher MS stages (MS^5^ and MS^6^) usually offered just one or two additional fragments to the whole spectral tree (e.g., FAICAR, SAdo, SAICAr). The loss of intensity observed in increasing MS stages caused higher noise levels, making the spectra less reliable for interpretation. Nevertheless, even these spectra could help with the identification of unknown molecules in mass spectral databases. One of the greatest advantages of spectral trees is based on the comparison of substructural information of similar compounds—precursor ion fingerprinting (PIF)—assuming that similar compounds share identical substructures and the uniqueness of a compound is given by the way in which substructures combine [21–23].

Generally, the instability of the majority of ribotides and ribosides was observed as a result of ion-source fragmentation. MS^2^ fragments were visible in full MS scans of nearly all compounds, most often not exceeding 5% of the intensity of a molecular ion. The intensity of some MS^2^ fragments in full MS scans reached 17, 20, and 540% of the intensity of the molecular ions for GAr, SAdo, and FGAr, respectively. GAr was the first compound to be identified with such profound in-source fragmentation and was chosen for the optimization of the measurement conditions. The initial parameters of the electrospray ionization (ESI) source were a 3 kV spray voltage, 300°C source heater temperature, 350°C capillary temperature, and 60% S-Lens RF level. ESI measurements under various conditions were performed (spray voltage 2.5 and 2 kV; heater and capillary temperatures lowered to 250 and 300°C, respectively; and S-lens parameters of 10, 20, and 40%) to reduce the ion-source fragmentation of fragile ions. No change in these parameters resulted in significantly better stability (see S2–S4 Figs). For compounds suffering from ion-source fragmentation and with low molecular ion intensity, the strategy for obtaining MS^n^ spectra was modified. MS^2^ analysis of the ion-source fragments (corresponding to MS^3^) belonging to the compound in the full MS spectrum was performed.

**Fig 3:**
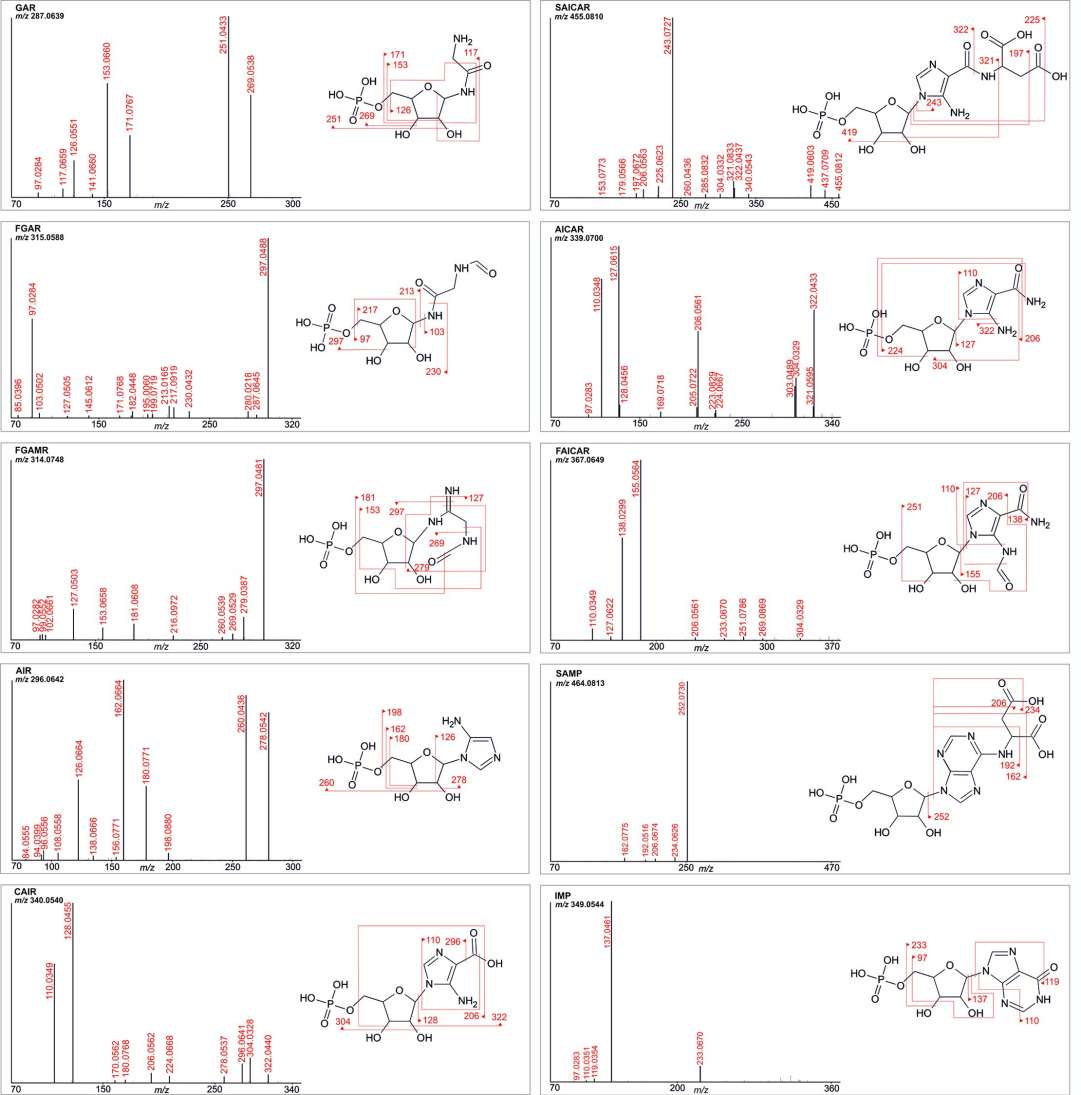
MS^2^ fragmentation spectra of PDNS ribotides. The structure of the fragmented compound is shown next to every spectrum, and up to six of the most intense fragments are depicted in the structure.

Ribotides showed considerable variability in their fragmentation patterns compared to ribosides (Fig 3). Losses of water or the phosphate group from ribose were the dominant events for the most intense fragments of the first part of the PDNS pathway (up to AIR). Other fragments appeared with the build-up of the purine ring, starting with CAIR and proceeding to consecutive PDNS intermediates. The most intense fragments of the ribotides of the second part of the pathway originated from the breaking of the N-glycosidic bond and the loss of an amino group from the base, similar to their dephosphorylated analogues.

All the ribosides shared common fragmentation behavior when the MS^2^ spectra were compared (see S5 Fig). The most intense fragment ion always belonged to the part of the base forming the purine ring, suggesting that the N-glycosidic bond had the highest susceptibility to breaking. In some cases (FAICAr and SAdo), the formation of this fragment greatly predominated, and all the other fragments were found at below 1% of the intensity of the dominant fragment ion but still above the lowest detectable signal. The majority of the second-most-intense fragment ions were represented by a basic part that had lost either the amino or a hydroxyl group. The loss of one or more molecules of water from the ribosidic part of nucleosides was characteristic of the subsequent fragments.

**Fig 4:**
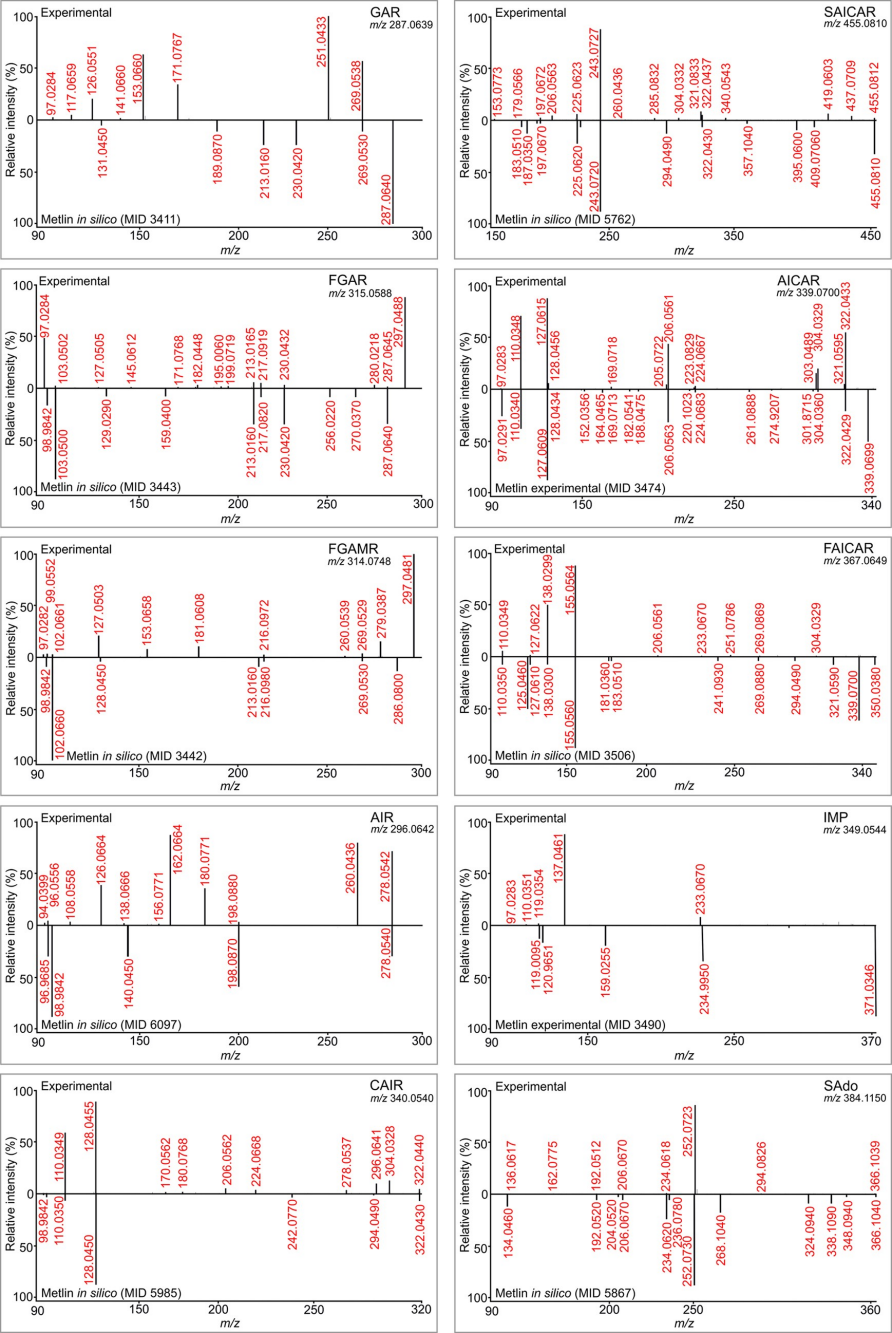
MS^2^ spectral comparison. PDNS metabolites present in the Metlin database (either as *in silico* or experimental fragmentation spectra) were compared with experimental spectra acquired in our study. The mass range of fragmentation spectra from the database was adjusted to the mass range of the spectra from our study.

To identify unknown structures, free online databases of fragmentation spectra can be used in advance. In the case of PDNS metabolites, databases (Metlin, HMDB, mzCloud) [24] do not contain experimental fragmentation spectra, just *in silico* predictions. Moreover, *in silico* fragmentation spectra are available for PDNS ribotides but not for ribosides. The MS^2^ spectra of the PDNS ribotides acquired in our study were compared with *in silico* spectra provided by the Metlin database (Fig 4), except for AICAR and IMP, which both have only experimental spectra in the database.

Generally, the experimental spectra match the *in silico* predictions by roughly one-third. Metlin *in silico* spectra generated by a machine learning approach are characterized by prioritizing low-molecular-weight ions (less than *m/z* 100) under all three different fragmentation sets of conditions tested in positive mode (10, 20, and 40 eV). In contrast, our experimental spectra offered many ions higher than *m/z* 100; the low-molecular-weight ions were suppressed because of the mechanism of CID fragmentation. The diversity of the ions in the experimental spectra can be attributed to combined sites of fragmentation leading to a particular ion. For example, in the MS^2^ experimental spectrum of GAR (Fig 4), the most significant fragments originate from the loss of one or two molecules of water from ribose (*m/z* 251 and 269) or the loss of one or two molecules of water from ribose in combination with the loss of the phosphate group (*m/z* 153 and 171). Of these four most significant ions, the *in silico* MS^2^ spectrum of GAR (MID 3411) offers just the ion of *m/z* 269, suggesting that only one fragmentation site is preferred for *in silico* fragment prediction. This assumption could explain the difference observed between the *in silico* and experimental spectra of the PDNS metabolites. Another reason for the spectral disagreement could arise from the existence of predicted ions that would probably not be generated in a given ionization mode (in our case, positive). An example is the ion of *m/z* 98.9842, annotated as a positively charged phosphate group (using MassFrontier for annotation). This ion appears in all the *in silico* spectra of PDNS ribotides but is unlikely to arise in positive ESI mode. *In silico* fragmentation approaches often use experimental spectra of commercially available compounds in the prediction process of commercially unavailable compounds (e.g., PDNS metabolites). AICAR is an example of a commercially available PDNS metabolite whose experimental spectra are present in the Metlin database. Generally, a greater number of equivalent fragment ions were found when comparing the experimental and predicted spectra of metabolites that structurally resemble the AICAR metabolite from the second part of PDNS. In contrast, the fragmentation spectra of metabolites from the first part of PDNS (GAR, FGAR) differ significantly from the *in silico* predictions. Despite the known limitations, *in silico* spectra can be helpful in the process of the identification of unknown structures or confirmation of supposed structures.

**Fig 5:**
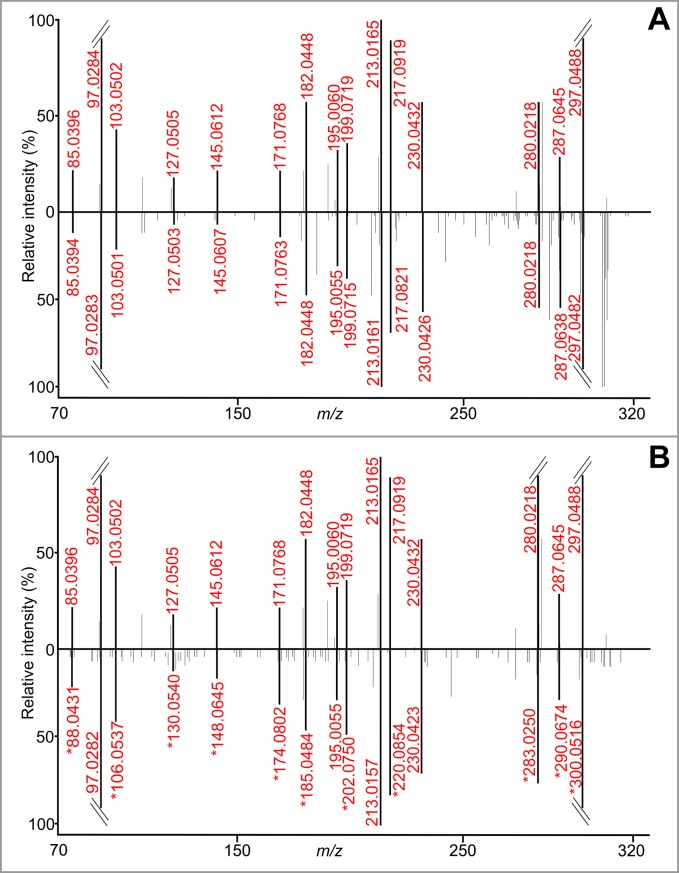
Confirmation of the identity of FGAR. The MS^2^ spectrum of synthesized FGAR was compared to the MS^2^ spectra of FGAR (A) and isotopically labeled FGAR-L (B), both of which were accumulated in CR-PFAS HeLa cells. In B, fragments with a mass shift of 3.0038 Da (because of the U-^13^C_2_, ^15^N labeling) are marked with an asterisk. The retention time of all compounds was 8.98 min.

The analysis of the HeLa cells enzymatically deficient in the individual steps of PDNS provided data for confirmation of the structures of the synthesized PDNS intermediates. The fragmentation patterns of the PDNS intermediates produced by cells were compared with those obtained by fragmentation analysis of the synthesized compounds. The HeLa cell lines (deficient and control) were cultivated in a glycine-free medium with the addition of isotopically labeled glycine. Glycine enters PDNS in the second enzymatic reaction catalyzed by a trifunctional GART enzyme, incorporating a glycine backbone into the forming purine structure. The PDNS pathway was stimulated by cultivating cells in purine-depleted media 24 hours before stable isotope labeling. Applying this procedure, we obtained naturally labeled PDNS intermediates serving as another means of confirmation of identity. Identity was confirmed by comparing the MS^2^ spectra of synthesized compounds to the MS^2^ spectra of these isotopically labeled metabolites on the basis of mass shift and considering the position of glycine incorporation and fragmentation. An example of the results of the mass shift experiment is shown in Fig 5. The majority of the PDNS metabolites detected in both cell lysates and growth media were found in the labeled forms. PRA was not detected as a result of its known chemical instability [16]. We were not able to detect FAICAR, probably because of the specific kinetic properties of ATIC [25]. ATIC is a bifunctional enzyme catalyzing the final transformylation of AICAR and cyclization of FAICAR to IMP. The spatial proximity of the two active sites, in which the formylation reaction favors a backward direction while the terminal cyclohydrolase is essentially unidirectional, makes FAICAR difficult to diffuse.

### Metabolic changes in edited HeLa cell line cultures

The regulation of PDNS has been studied since the 1950s. Enzyme kinetics experiments, as well as theoretical calculations, have proposed that only the first step of PDNS is limiting for the pathway and that no change in other steps has an impact on the overall flux [26–28]. AICAr and SAICAr are the only PDNS intermediates detectable in the bodily fluids of healthy humans by current analytical technologies [10, 17, 29, 30]. These observations imply that other regulatory places might exist within the PDNS pathway.

**Fig 6:**
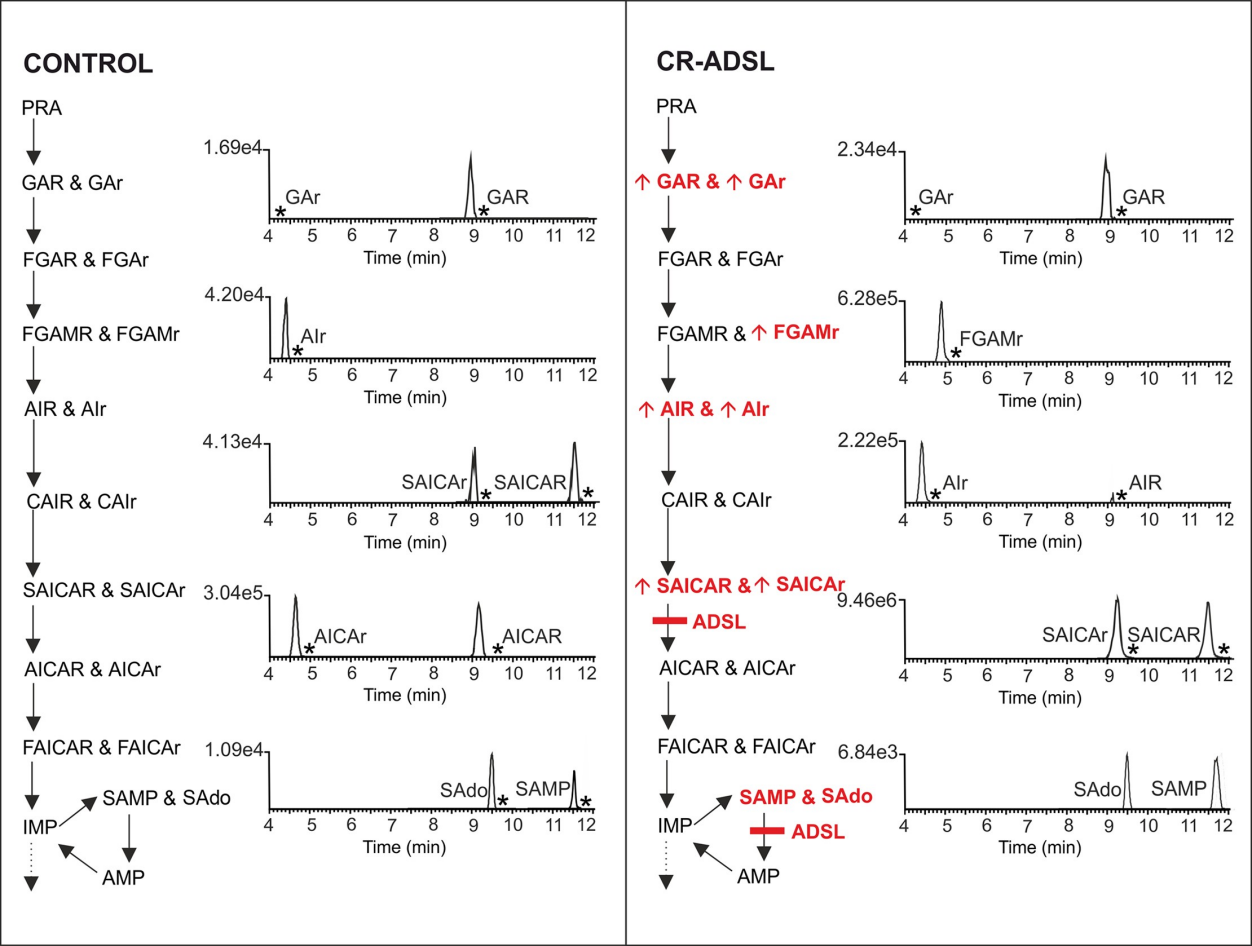
Ribotides and ribosides detected in cell lysates of ADSL-deficient and control HeLa cells. The deficient cell line is graphically represented by a PDNS pathway with an enzyme block marked by a red rectangle. Accumulating metabolites are marked with red bold letters with an arrow. Cell lines were measured in hexaplicate, and the value of the unlabeled peak intensity given above the baseline represents the average of a particular metabolite. Chromatographic peaks of metabolites that were detected also in labeled form are shown as an asterisk. Note: The ionization efficiency of ribotides and ribosides is substantially different, so the responses of these compounds are not directly comparable.

The first knockout human cellular models of both known and potentially novel genetically determined defects of PDNS, published in 2016 by Baresova et al. [11], were used in the study. The aim was to model the situation in the bodily fluids of patients. All cells were transferred into purine-depleted medium for 24 hours prior to the experiment to stimulate PDNS. The HPLC-HRMS method was applied to analyze the accumulated PDNS metabolites in the cell lysates and growth media. Overall, six CRISPR-Cas9 genome-edited defective HeLa cell lines (CR-GART, CR-PFAS, CR-PAICS, CR-ADSL, CR-ATIC, and CR-HGPRT) and control HeLa cells were analyzed. All the ribotides and ribosides detected in control and ADSL-defective HeLa cells are listed in Fig 6 (see other defective cell lines in S6 Fig). The majority of the PDNS metabolites detected in both cell lysates were found in the labeled forms. In all the deficient cell lines, we were able to detect both intracellular and extracellular metabolites, except GART-deficient cells, because of the instability of the theoretically expected accumulating PRA, which has a half-life of 5 s under physiological conditions [15, 16]. The accumulation of intermediate ribotides of a particular defective enzyme causes a shift in the equilibria of upstream enzymatic reactions. Because of this effect, we also observed an accumulation of intermediates of PDNS enzymes up to five steps prior to the defective step. In these cases, we usually did not observe an accumulation of metabolites one by one in the opposite direction preceding the main enzyme block. Not all such intermediates were detected because of the chemical instability of the metabolite, differing enzyme kinetics across PDNS, and different ionization efficiencies of PDNS intermediates. The fragmentation method used generally favors ribosides over the more negatively charged ribotides. This might be the reason why ribotides are not detected in cells in some cases but the dephosphorylated analogues of the ribotides are present. Some of the dephosphorylated forms of PDNS intermediates were detected in both cell lysates and media. This is a usual route for defective cells in both the *de novo* and salvage purine pathways to eliminate toxic accumulated intermediates via equilibrative nucleoside transporters. These suggested mechanisms hold for purine salvage pathway defects [31] and are also supported by biochemical observations in patients deficient in ADSL and ATIC, where only ribosidic forms of substrates are detected in the body fluids of the patients [7, 32]. These mechanisms are of the utmost clinical importance because extracellular nucleosides are used as diagnostic biomarkers of these diseases [31]. Human equilibrative transporters possess highly different catalytic activities towards their substrates, and these substrates compete for the active site [33]. Additionally, there is an unknown specificity of purine nucleoside phosphorylase towards our metabolites. These facts make the interpretation of the levels of both natural and labeled PDNS metabolites difficult, and Fig 6 represents just an illustrative view.

In the cytoplasm of control HeLa cells cultured in purine-free medium (Fig 6), AICAR and SAICAR were detected, as were their dephosphorylated forms, which is in agreement with previous reports [29, 30]. Moreover, we were able to detect natural GAR/r and AIr in control cell lysates for the first time, suggesting higher sensitivity of the method. PDNS is considered to be a pathway with an initial rate-limiting step regulating the overall flux, leading to an assumption that its substrates are at low and mutually related concentrations. Thus, their detection probably depends on the ionization efficiency of the individual PDNS intermediates. The experiments could be influenced by the differing viability of the individual defective cell lines used, providing highly variable numbers of cells in each cultivation flask. There is a single study reporting the partial detection of intermediates at trace levels by radiolabeling with autoradiography [5]. The purine salvage metabolites SAMP and SAdo were also detected. Two ribosides (AICAr and AIr) were excreted into the growth media of the control cells.

PFAS-deficient cells (CR-PFAS cells) accumulated FGAR and its riboside, which was also detected in growth media. Most likely, accumulating FGAR causes a shift in equilibrium for trifunctional GART, resulting in a detectable amount of GAR. FGAR and GAR were also detected as isotopically labeled analogues.

The accumulation of AIR in CR-PAICS cells was previously described in Chinese hamster ovary cells. These cells deficient in PAICS activity also accumulated a second compound that the authors were not able to identify [8]. In our experiment, we detected three other ribotides preceding the enzymatic block in addition to the expected accumulation of AIR. The abundant ribotides were excreted into growth media in dephosphorylated forms. This observation suggests a shift in the equilibria of enzymatic reactions up to the PFAS enzyme, as FGAR and FGAr were the last detectable metabolites.

The deficiency of the bifunctional enzyme ADSL is characterized by the presence of the succinylpurines SAICAr and SAdo in bodily fluids [34]. In our experiment, CR-ADSL cells accumulated SAICAR and SAICAr; however, SAMP and SAdo were detected but not accumulated compared to the controls. This behavior could be explained by the experimental set-up, where the purine-free medium forces cells to use PDNS extensively. The purine nucleotide cycle, however, might not be used because of the low energy demands of the cultured cells, which do not possess high proliferation. Two other PDNS intermediates were detected in the cell lysates—AIR and GAR. These metabolites probably originate from the shifted equilibria of enzymes preceding the ADSL blockage. AIr, FGAMr, and GAr were other ribosides detected in cells. SAICAr, AIr, and FGAMr were found in growth media. The accumulation of AIR/AIr in cells and growth media is in accordance with the findings from ADSL-deficient Chinese hamster ovary cells (AdeI), where the authors detected the accumulation of AIR [8].

In the one patient suffering from ATIC deficiency reported so far, a massive accumulation of AICAr, SAICAr, and SAdo in bodily fluids was detected [7]. CR-ATIC cells accumulated AICAR and SAICAR. Corresponding ribosides were also found in growth media. SAMP and its riboside SAdo were not detected.

Patients with partial or complete HGPRT deficiency (Kelley-Seegmiller syndrome and Lesch-Nyhan syndrome, respectively) are known to have increased levels of AICAR in their erythrocytes [35] and AICAr in their urine [36]. As mentioned previously, erythrocytes lack an intact PDNS pathway but are capable of metabolizing exogenous ribosides to their corresponding mono-, di-, and triphosphates and accumulating the metabolites [35]. Several studies reported increased AICAR contents in the brains of HGPRT-deficient mice [37, 38]. We detected AICAR in cell lysates and AICAr in both the cell lysates and media of CR-HGPRT cells. The bifunctional enzyme ATIC, responsible for the last two steps of the pathway, is strongly inhibited by xanthosine-5'-monophosphate, which is accumulated in patients with Lesch-Nyhan syndrome because of the massive flux to uric acid production [39]. Another possible hypothesis for the accumulation of AICAR is enhanced histidine biosynthesis in HGPRT patients; however, there are no experimental data to support this hypothesis [38, 40]. As a result of shifted enzyme equilibria, labeled SAICAR was also detected.

## Conclusion

In this study, we provide the first report of a comprehensive mass spectrometric fragmentation analysis of synthesized PDNS intermediates and their dephosphorylated analogues. HRMS fragmentation was possible in the third to sixth stages, and the spectra were compared with *in silico* spectra present in free online databases. The data acquired allowed the development of the method for the detection of these compounds in HeLa cells deficient in the individual steps of PDNS. Our method is applicable in various areas of PDNS research, such as studying cell cycle/purinosome formation. The detection of PDNS intermediates (ribotides) can be valuable in enzyme kinetics studies since numerous compounds are claimed to be inhibitors of this metabolic pathway. Medical applications include the development of diagnostic methods for known/putative inherited metabolic disorders of PDNS and understanding their pathobiochemistry.

## Supporting information

S1 Table List of initial concentrations of PDNS metabolites that were subjected to HRMS^n^ fragmentation analysis. (TIF)

S1 Fig Fragmentation spectral trees of GAR/r, FGAR/r, FGAMR/r, AIR/r, CAIR/r, SAICAR/r, AICAR/r, FAICAR/r, IMP, SAdo and SAMP. All ribotides are marked with a capital R in the name; ribosides are marked with a lowercase r. Every *m/z* assigned with accurate mass in the spectra (in red) belongs to the fragmented structure. The other *m/z* represent coeluting compounds or masses belonging to fragmented structures that could not be identified using the given procedure.
(PDF)

S2 Fig An example of in-source fragmentation. MS^2^ spectra of GAr measured using decreasing spray voltages: 3 kV (A), 2.5 kV (B), and 2 kV (C). Intensities of the depicted fragments do not significantly change with altered conditions.
(TIF)

S3 Fig An example of in-source fragmentation. MS^2^ spectra of GAr measured using increasing S-lens parameters: 10% (A), 20% (B), and 40% (C). Intensities of the depicted fragments do not significantly change with altered conditions.
(TIF)

S4 Fig An example of in-source fragmentation. MS^2^ spectra of GAr measured using different temperatures of the heater (H) and capillary (CA): H 300°C, CA 350°C (A); H 300°C, CA 300°C (B); and H 250°C, CA 250°C (C). Intensities of the depicted fragments do not significantly change with altered conditions.
(TIF)

S5 Fig MS^2^ fragmentation spectra of PDNS ribosides. The structure of the fragmented compound is shown next to every spectrum, and up to six of the most intense fragments are depicted in the structure.
(TIF)

S6 Fig Ribotides and ribosides detected in cell lysates of PFAS-, PAICS-, ATIC- and HGPRT-deficient HeLa cells. Deficient cell lines are graphically represented by a PDNS pathway with an enzyme block marked by a red rectangle. Accumulating metabolites are marked with red bold letters with an arrow. Cell lines were measured in hexaplicate, and the value of the unlabeled peak intensity given above the baseline represents the average of a particular metabolite. Chromatographic peaks of metabolites that were also detected in labeled form are shown with asterisks. Note: The ionization efficiency of ribotides and ribosides is substantially different, so the responses of these compounds are not directly comparable.
(TIF)
