# Supplementary figures and images for: Mass spectrometric analysis of purine de novo biosynthesis intermediates
Source: PLoS One. 2018 Dec 10;13(12):e0208947. doi: 10.1371/journal.pone.0208947 (PMC6287904; doi:10.1371/journal.pone.0208947)

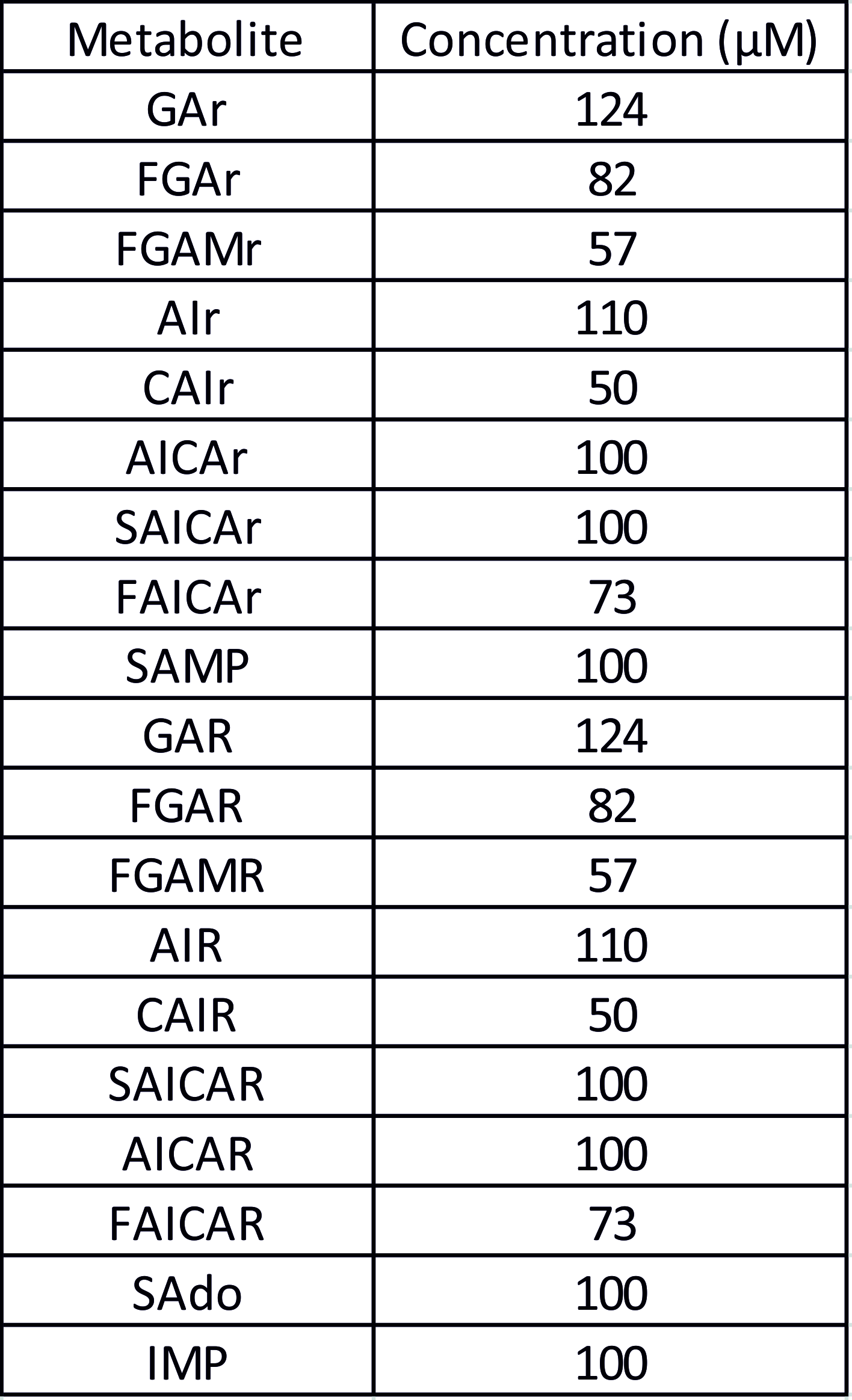

Supplement: S1 Table — (TIF) [file pone.0208947.s001.tif]

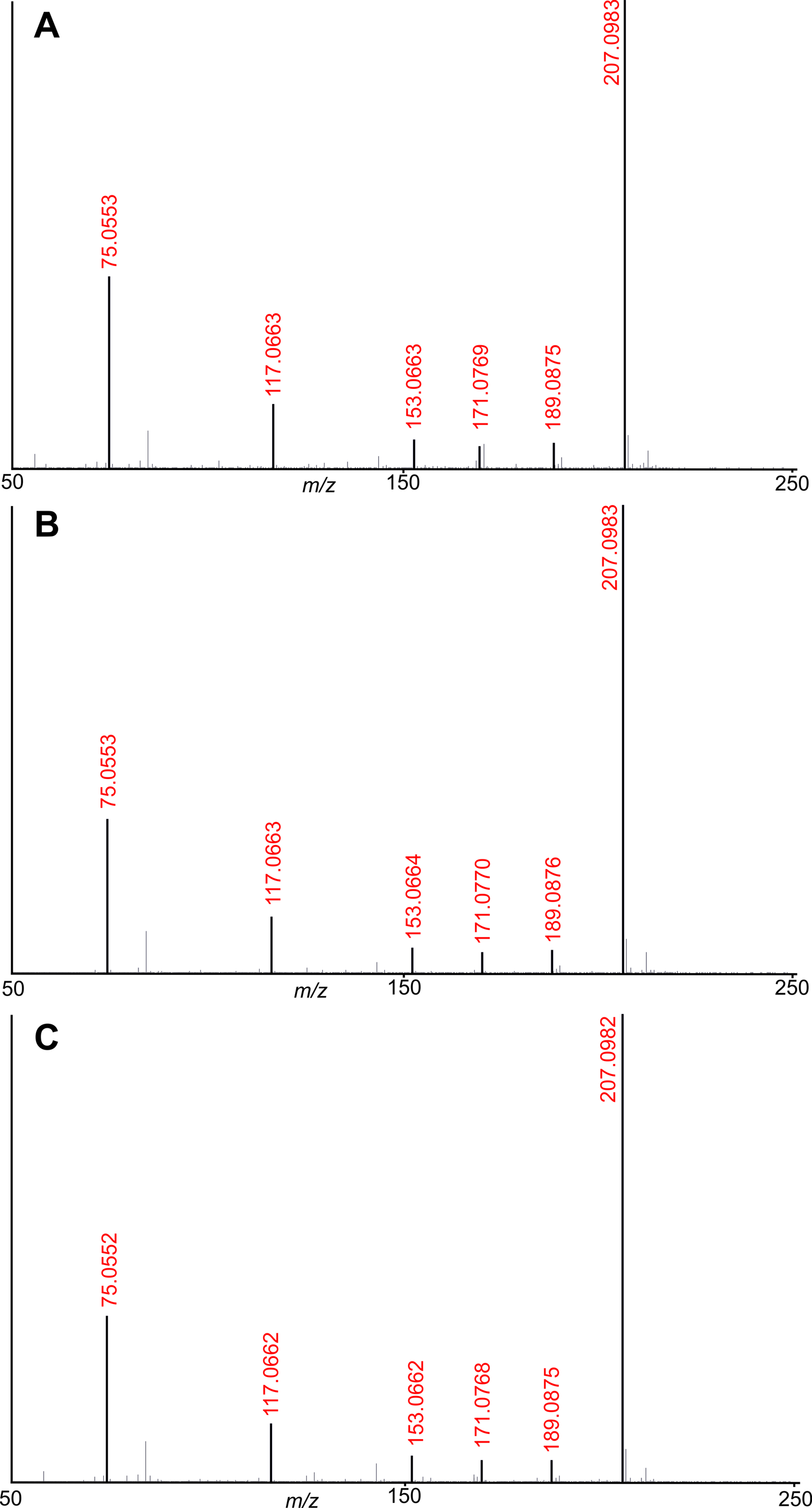

Supplement: S2 Fig — MS2 spectra of GAr measured using decreasing spray voltages: 3 kV (A), 2.5 kV (B), and 2 kV (C). Intensities of the depicted fragments do not significantly change with altered conditions. (TIF) [file pone.0208947.s003.tif]

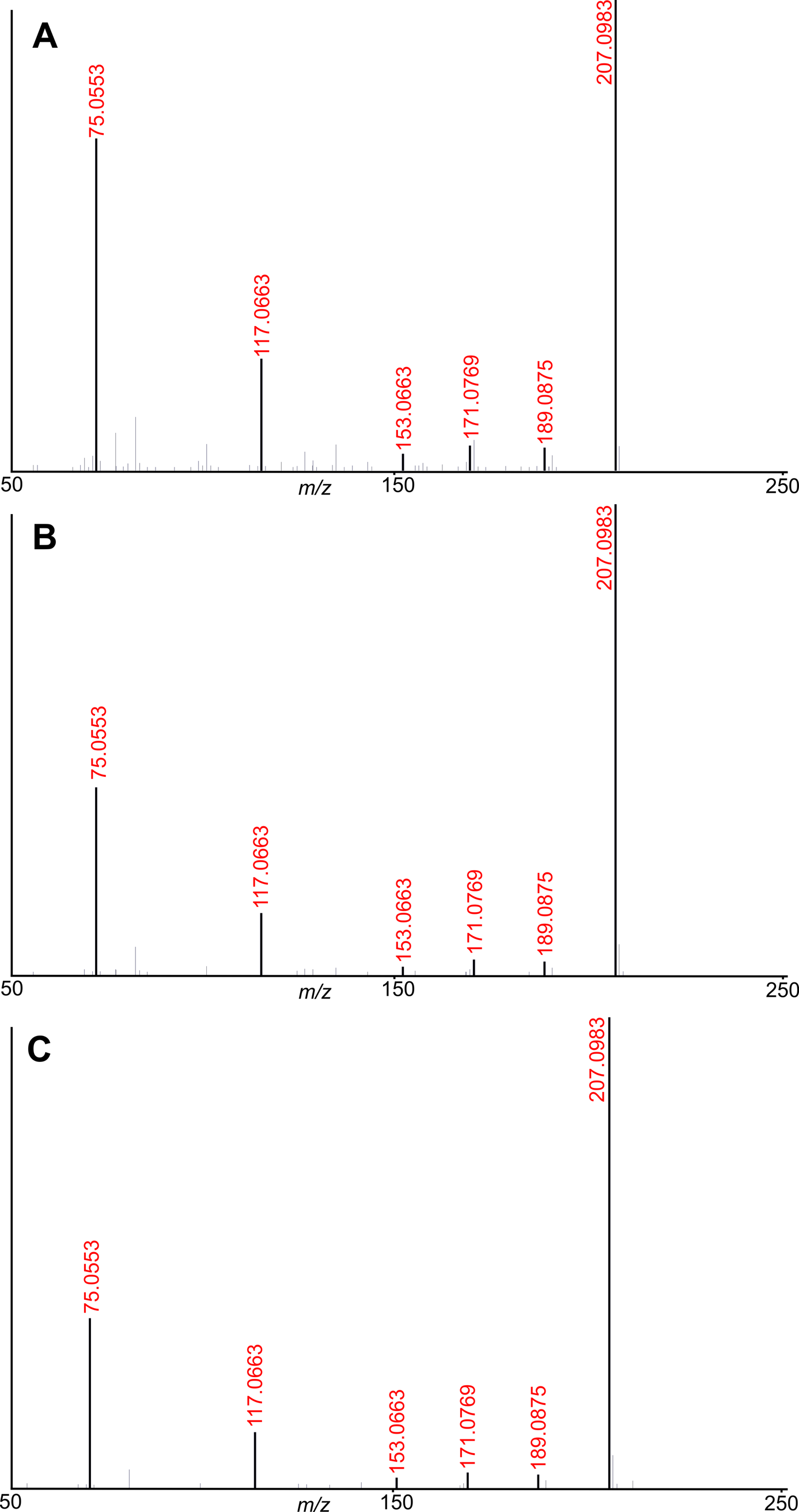

Supplement: S3 Fig — MS2 spectra of GAr measured using increasing S-lens parameters: 10% (A), 20% (B), and 40% (C). Intensities of the depicted fragments do not significantly change with altered conditions. (TIF) [file pone.0208947.s004.tif]

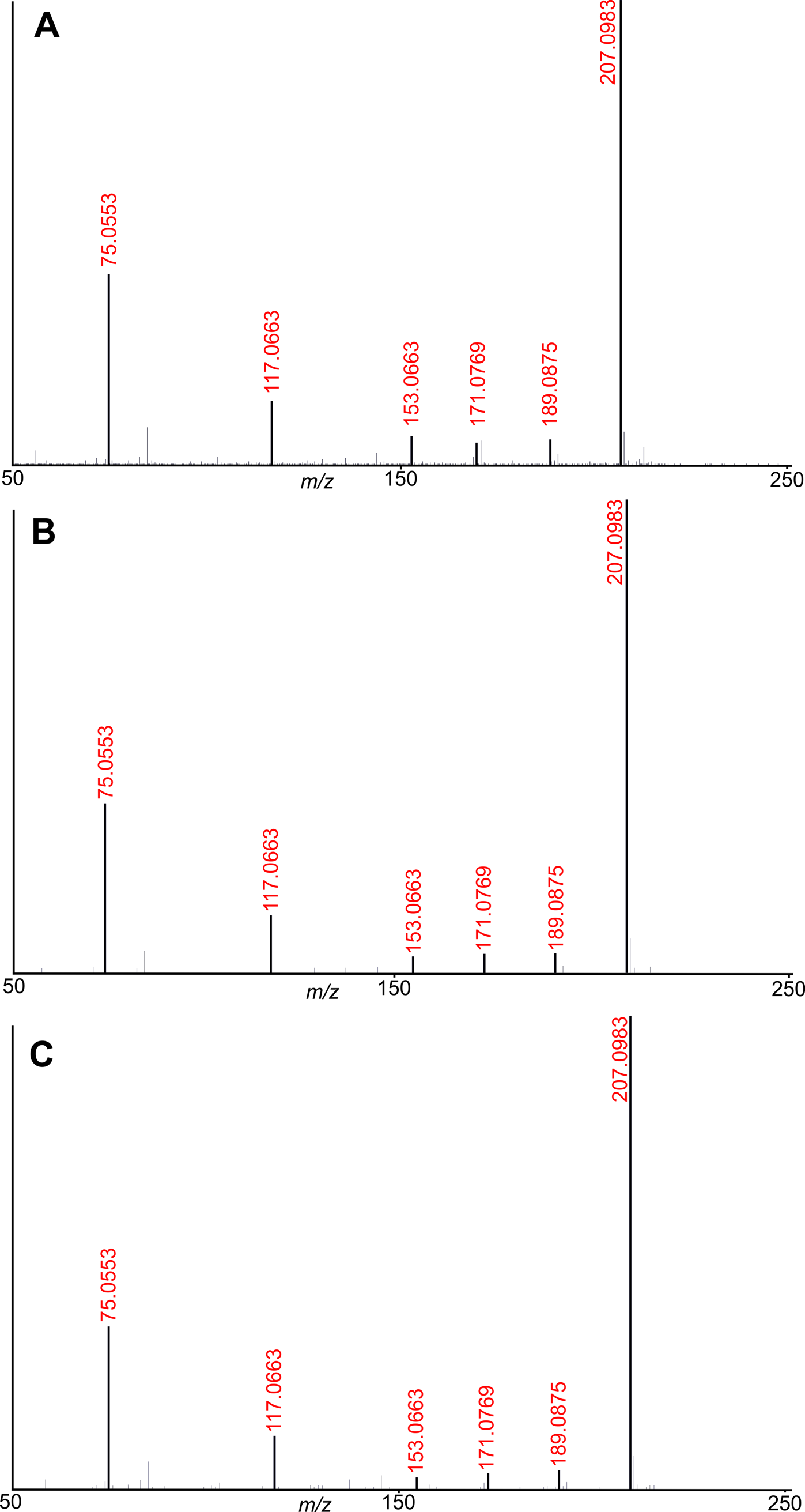

Supplement: S4 Fig — MS2 spectra of GAr measured using different temperatures of the heater (H) and capillary (CA): H 300°C, CA 350°C (A); H 300°C, CA 300°C (B); and H 250°C, CA 250°C (C). Intensities of the depicted fragments do not significantly change with altered conditions. (TIF) [file pone.0208947.s005.tif]

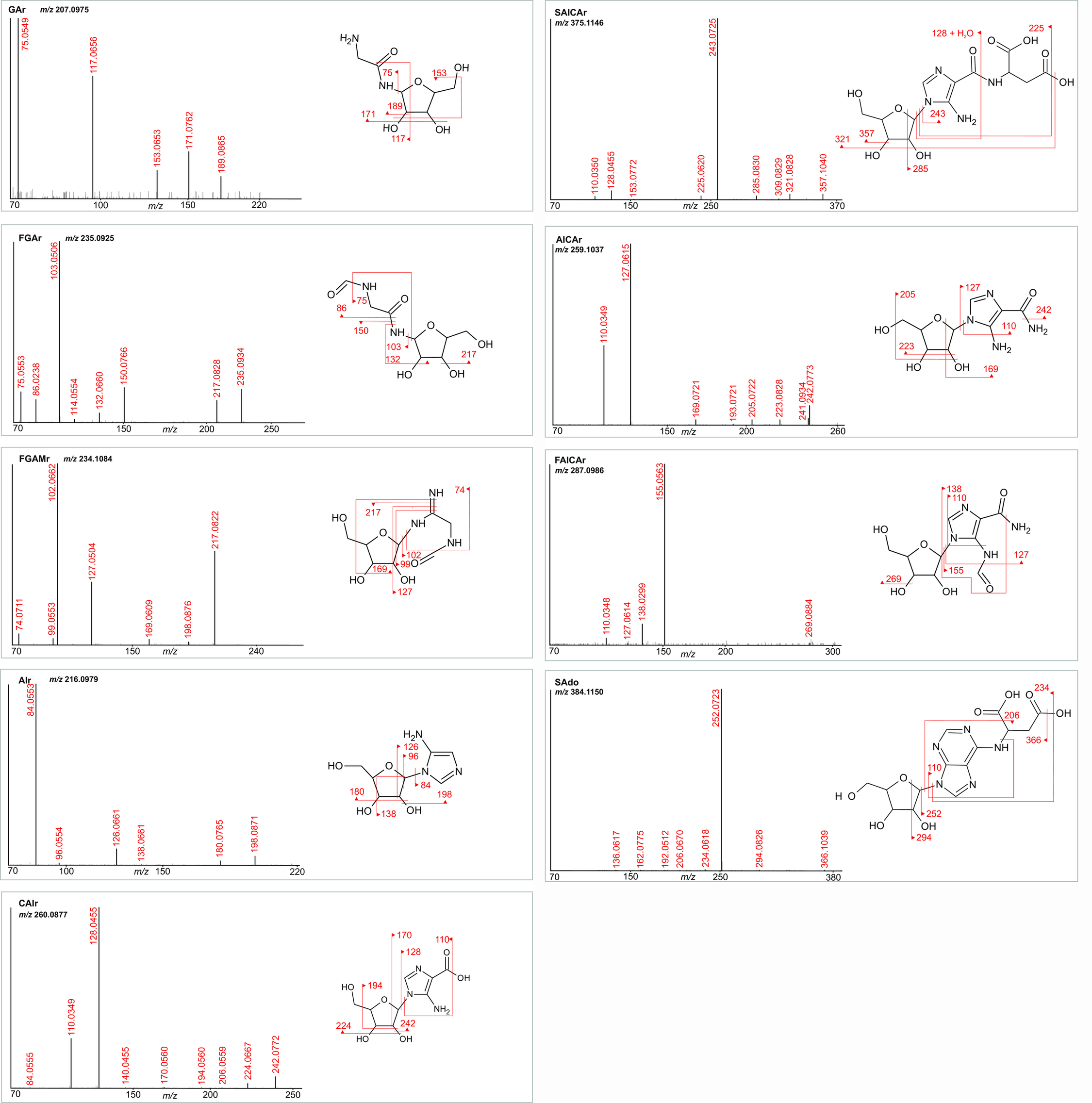

Supplement: S5 Fig — The structure of the fragmented compound is shown next to every spectrum, and up to six of the most intense fragments are depicted in the structure. (TIF) [file pone.0208947.s006.tif]

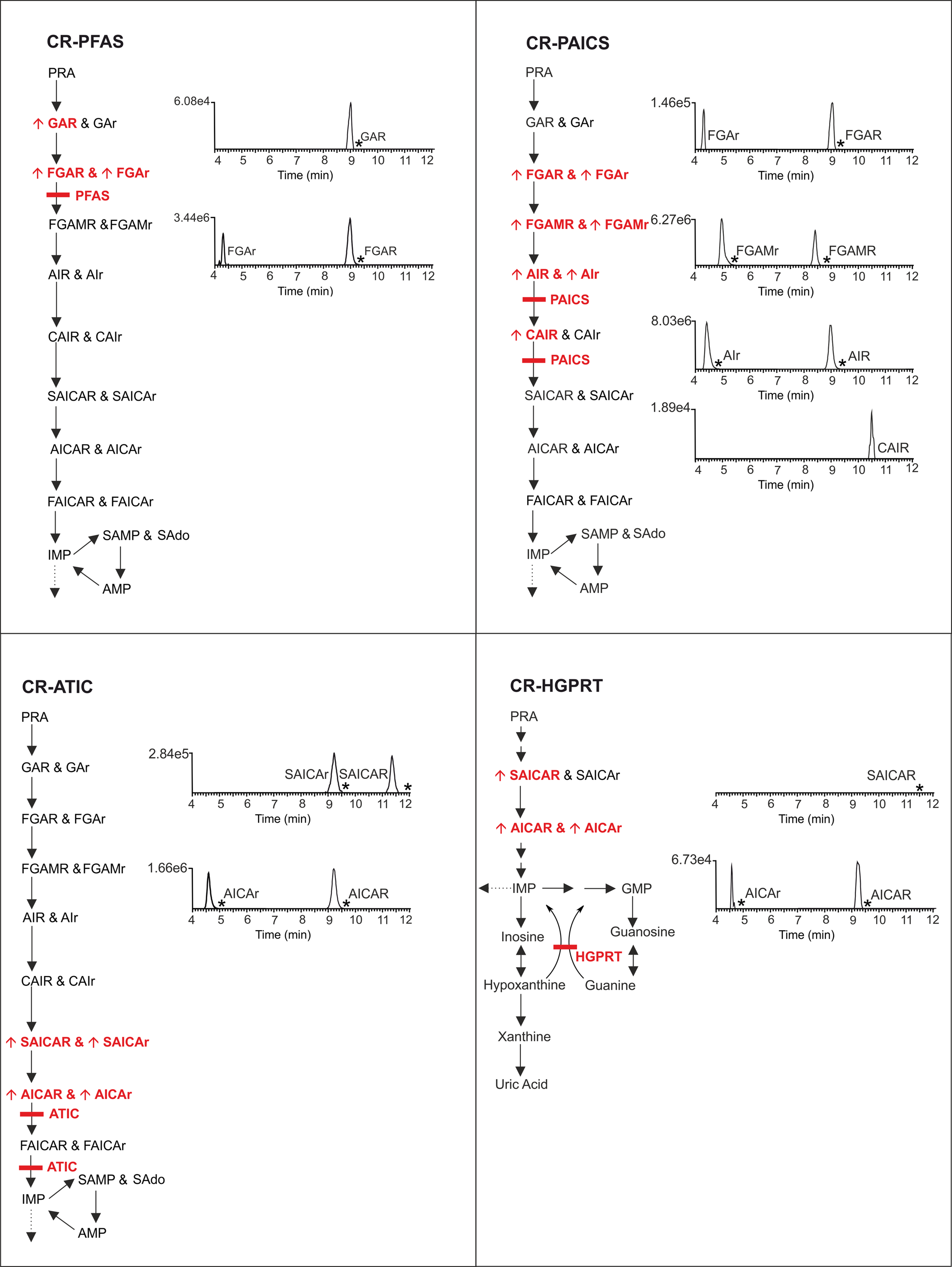

Supplement: S6 Fig — Deficient cell lines are graphically represented by a PDNS pathway with an enzyme block marked by a red rectangle. Accumulating metabolites are marked with red bold letters with an arrow. Cell lines were measured in hexaplicate, and the value of the unlabeled peak intensity given above the baseline represents the average of a particular metabolite. Chromatographic peaks of metabolites that were also detected in labeled form are shown with asterisks. Note: The ionization efficiency of ribotides and ribosides is substantially different, so the responses of these compounds are not directly comparable. (TIF) [file pone.0208947.s007.tif]
