# Supplementary material for: Mass spectrometric analysis of purine de novo biosynthesis intermediates
Source: PLoS One. 2018 Dec 10;13(12):e0208947. doi: 10.1371/journal.pone.0208947 (PMC6287904; doi:10.1371/journal.pone.0208947)
Supplement: S1 Fig — All ribotides are marked with a capital R in the name; ribosides are marked with a lowercase r. Every m/z assigned with accurate mass in the spectra (in red) belongs to the fragmented structure. The other m/z represent coeluting compounds or masses belonging to fragmented structures that could not be identified using the given procedure. (PDF) [file pone.0208947.s002.pdf]

GAr

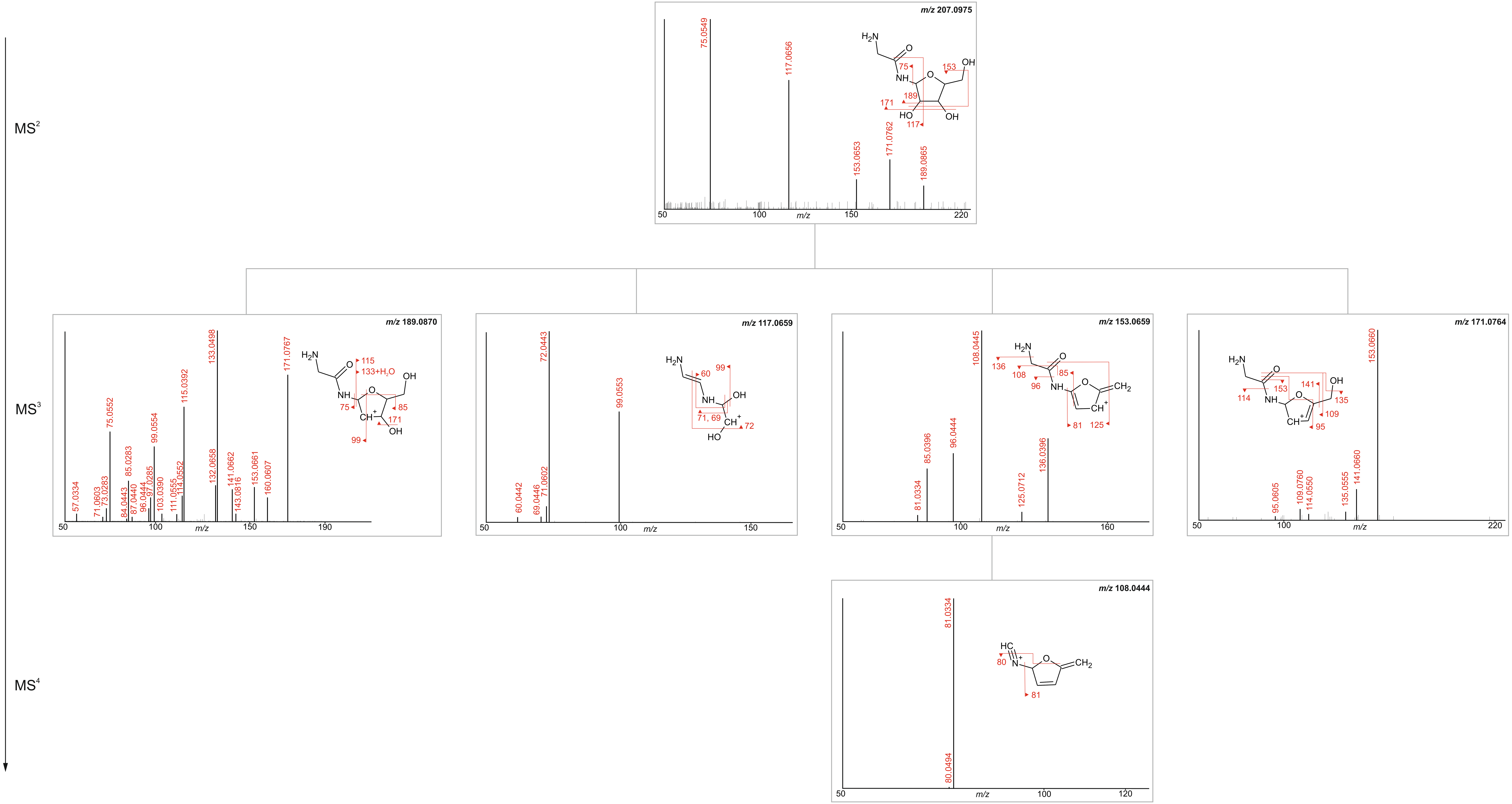
MS2
MS3
MS4
m/z 207.0975
75.0549
117.0656
153.0653
171.0762
189.0865
m/z 189.0870
57.0334
71.0603
73.0283
75.0552
84.0443
85.0283
87.0440
96.0444
97.0285
99.0554
103.0390
111.0555
114.0552
115.0392
132.0658
133.0498
141.0662
143.0816
153.0661
160.0607
171.0767
m/z 117.0659
60.0442
69.0446
71.0602
72.0443
99.0553
m/z 153.0659
81.0334
85.0396
96.0444
108.0445
125.0712
136.0396
m/z 171.0764
95.0605
109.0760
114.0550
135.0555
141.0660
153.0660
m/z 108.0444
80.0494
81.0334
m/z

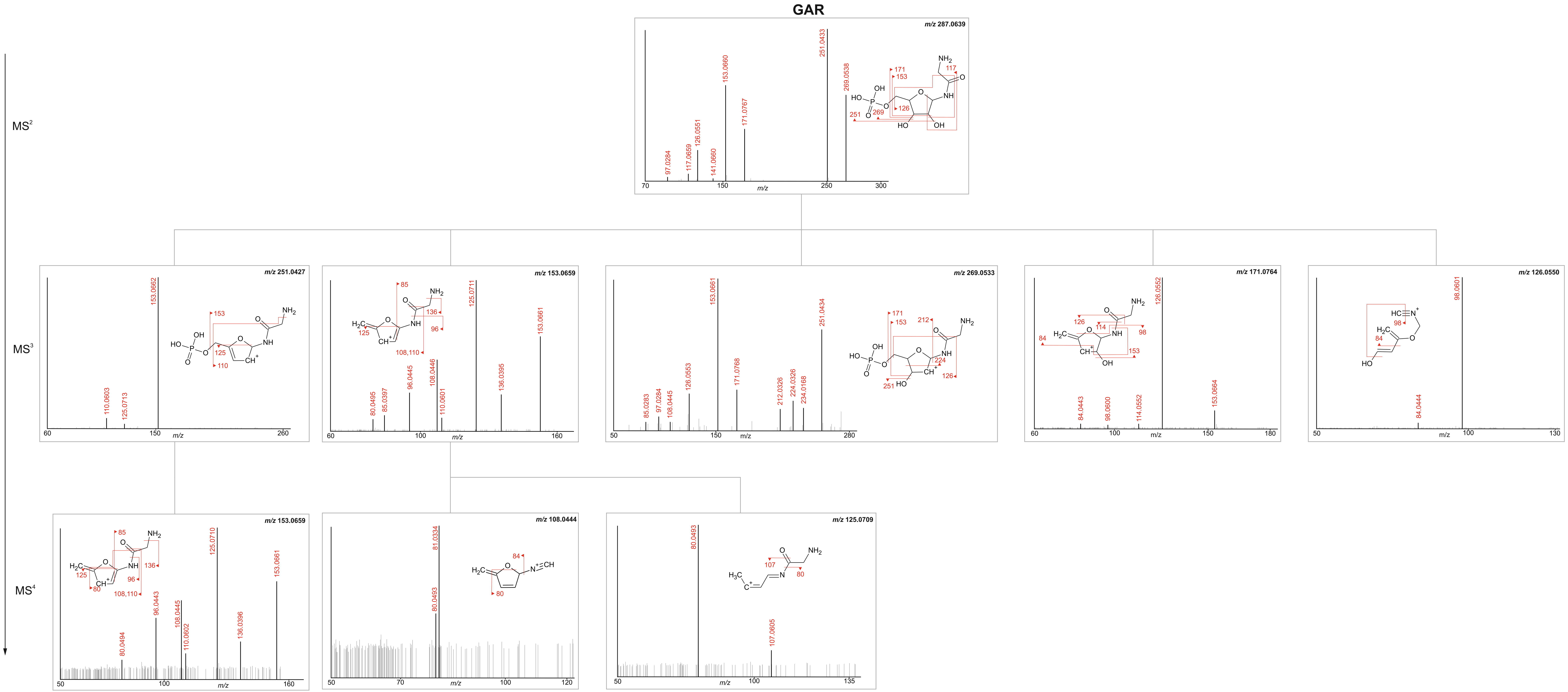
GAR
MS2
MS3
MS4
m/z 287.0639
97.0284
117.0659
126.0551
141.0660
153.0660
171.0767
251.0433
269.0538
m/z 251.0427
110.0603
125.0713
153.0662
m/z 153.0659
80.0495
85.0397
96.0445
108.0446
110.0601
125.0711
136.0395
153.0661
m/z 269.0533
85.0283
97.0284
108.0445
126.0553
153.0661
171.0768
212.0326
224.0326
234.0168
251.0434
m/z 171.0764
84.0443
98.0600
114.0552
126.0552
153.0664
m/z 126.0550
84.0444
98.0601
m/z 153.0659
80.0494
96.0443
108.0445
110.0602
125.0710
136.0396
153.0661
m/z 108.0444
80.0493
81.0334
m/z 125.0709
80.0493
107.0605
m/z

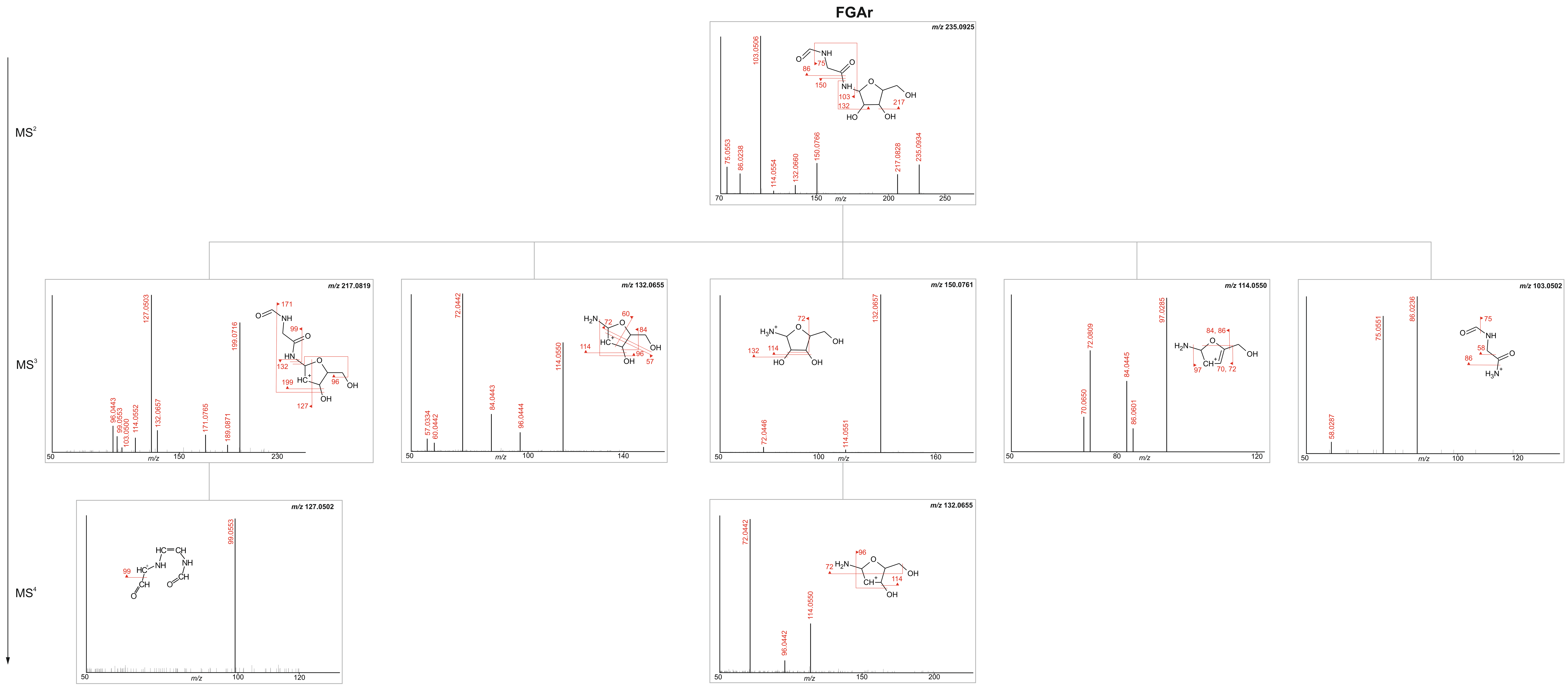

FGAr
MS2
MS3
MS4
m/z 235.0925
m/z 217.0819
m/z 132.0655
m/z 150.0761
m/z 114.0550
m/z 103.0502
m/z 127.0502
m/z 132.0655

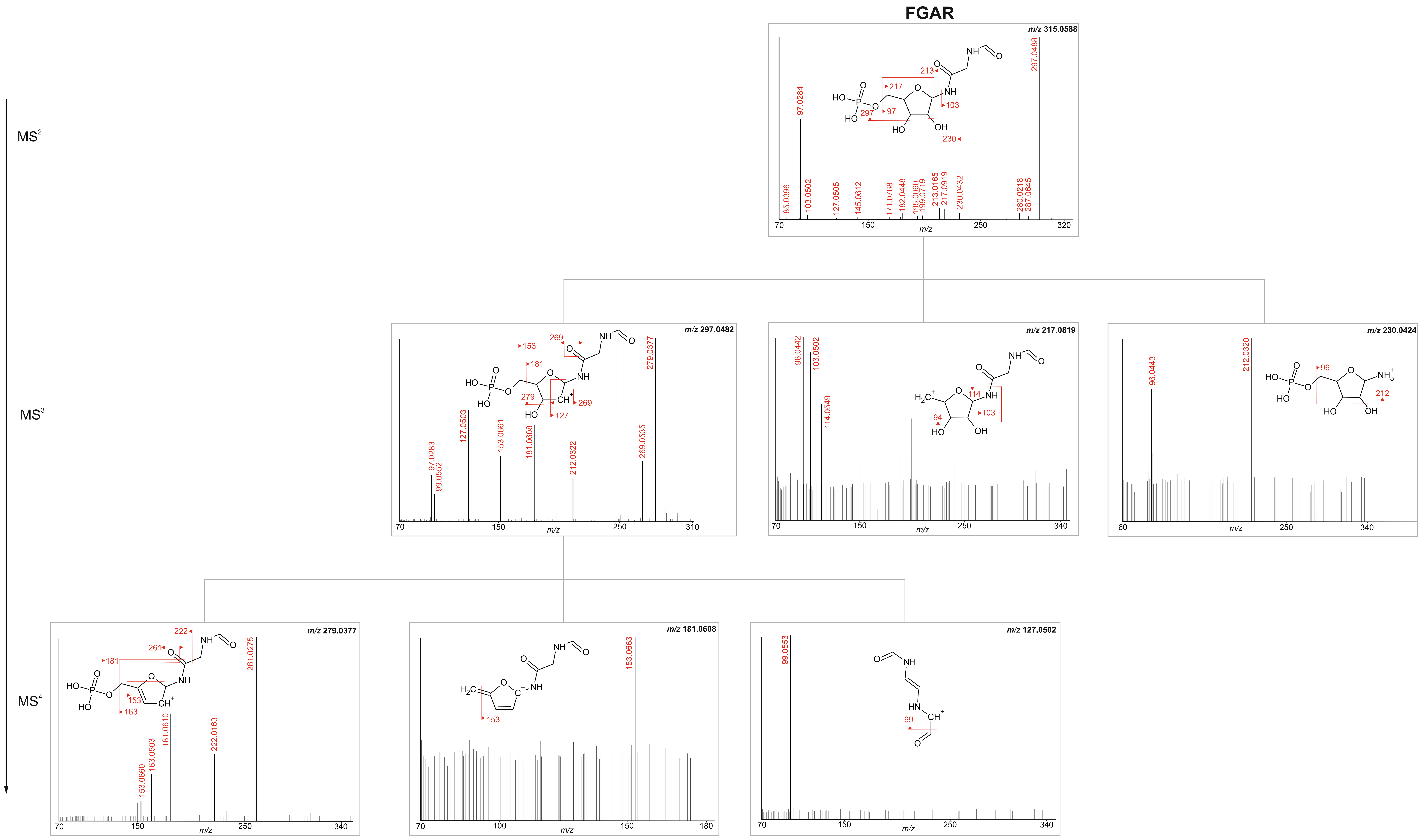
FGAR
MS2
MS3
MS4
m/z 315.0588
m/z 297.0482
m/z 217.0819
m/z 230.0424
m/z 279.0377
m/z 181.0608
m/z 127.0502
m/z
297.0488
97.0284
85.0396
103.0502
127.0505
145.0612
171.0768
182.0448
195.0060
199.0719
213.0165
217.0919
230.0432
280.0218
287.0645
279.0377
127.0503
153.0661
181.0608
97.0283
99.0552
212.0322
269.0535
96.0442
103.0502
114.0549
96.0443
212.0320
261.0275
153.0660
163.0503
181.0610
222.0163
153.0663
99.0553

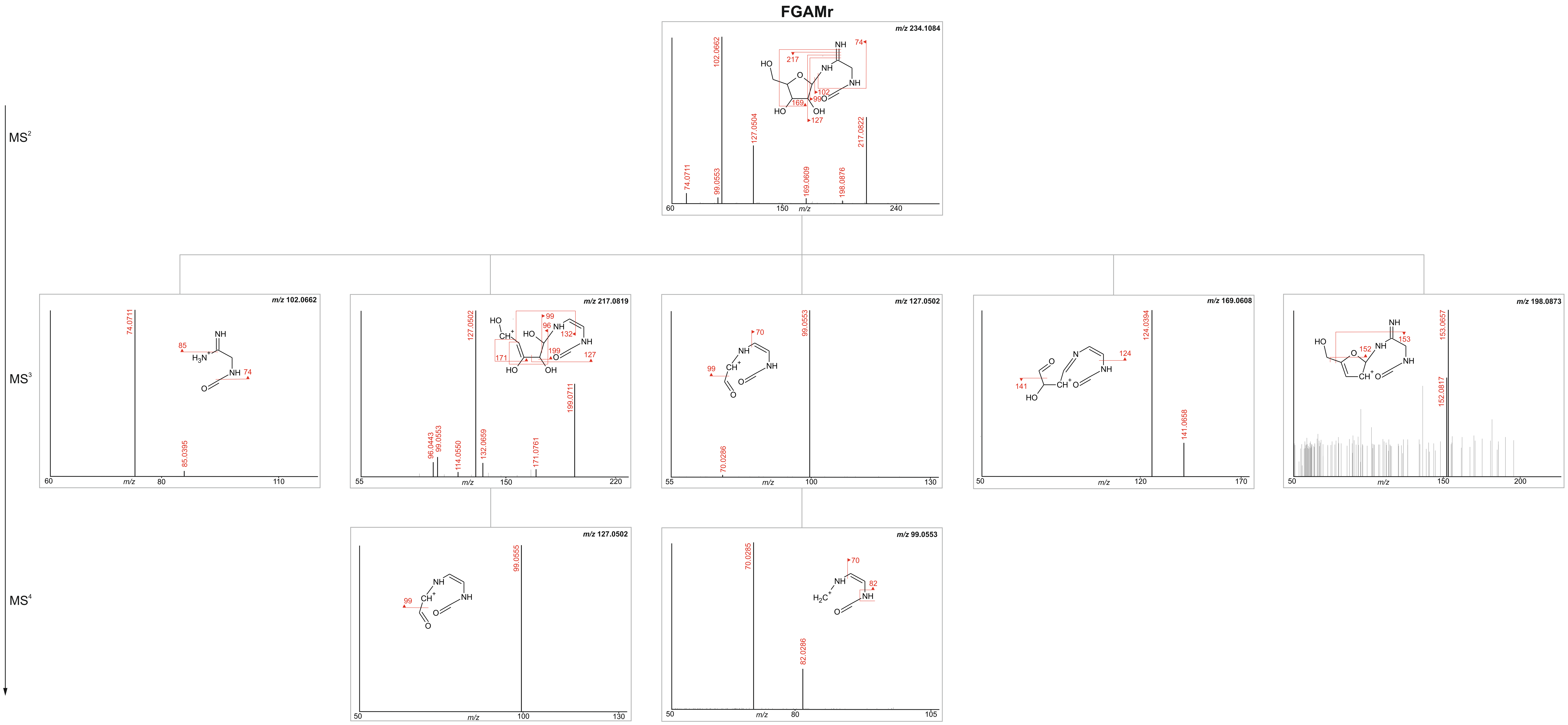
FGAMr
MS2
MS3
MS4
m/z 234.1084
m/z 102.0662
m/z 217.0819
m/z 127.0502
m/z 169.0608
m/z 198.0873
m/z 127.0502
m/z 99.0553
m/z

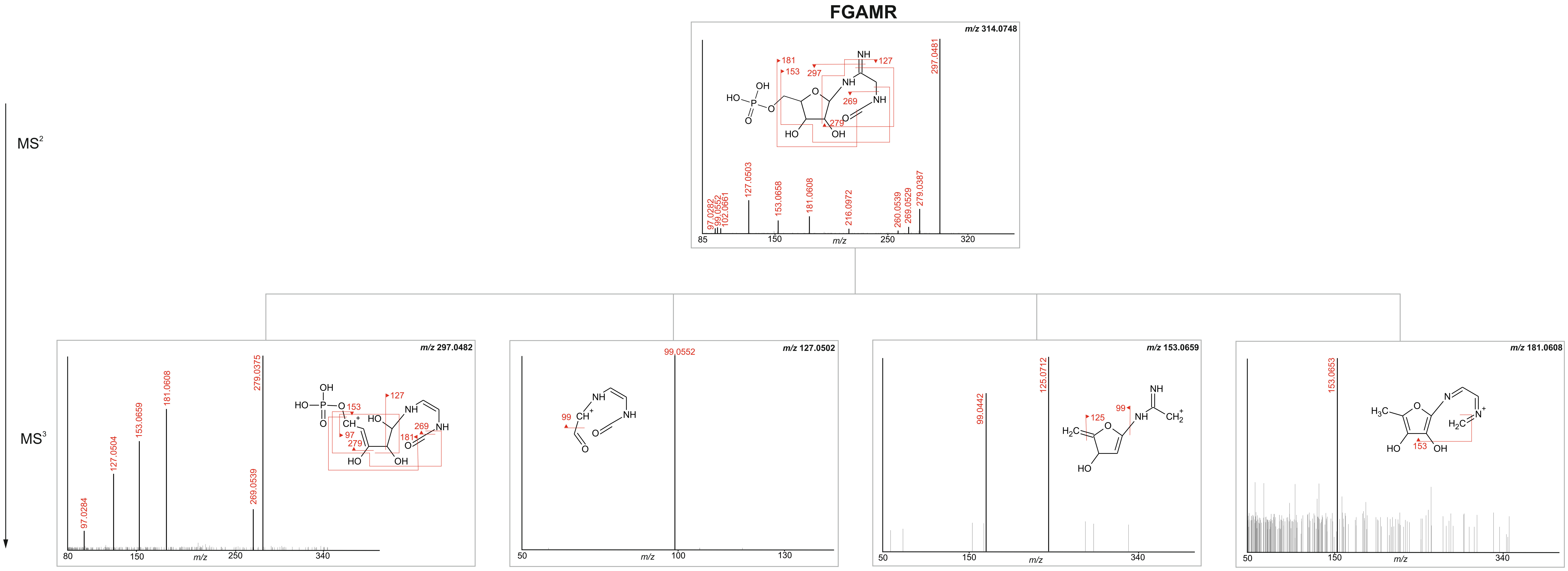

FGAMR
m/z 314.0748
MS2
MS3
m/z 297.0482
m/z 127.0502
m/z 153.0659
m/z 181.0608
97.0282
99.0552
102.0661
127.0503
153.0658
181.0608
216.0972
260.0539
269.0529
279.0387
297.0481
97.0284
127.0504
153.0659
181.0608
269.0539
279.0375
99.0552
99.0442
125.0712
153.0653
m/z

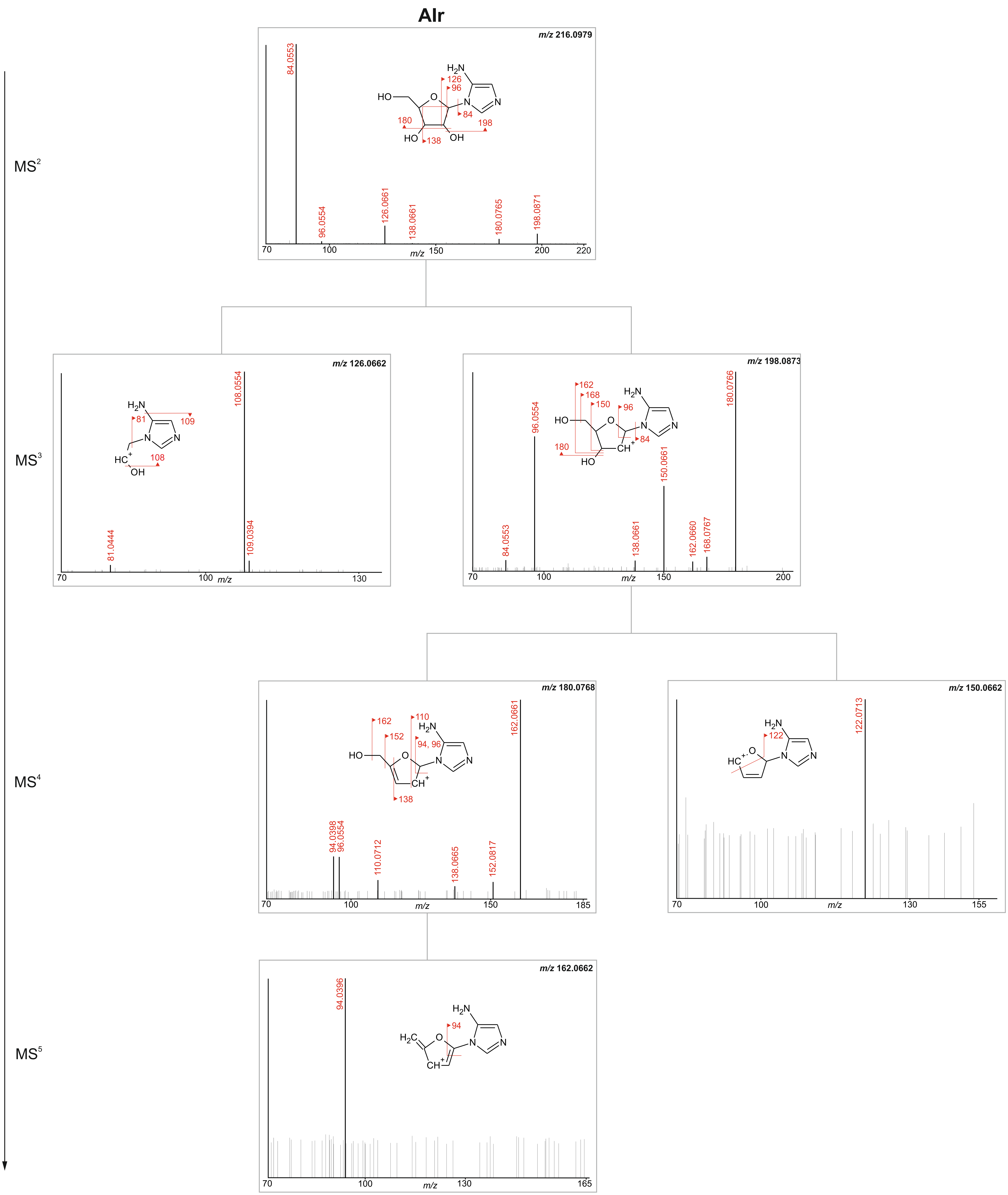
AIr
MS2
MS3
MS4
MS5
m/z 216.0979
84.0553
96.0554
126.0661
138.0661
180.0765
198.0871
m/z 126.0662
81.0444
108.0554
109.0394
m/z 198.0873
84.0553
96.0554
138.0661
150.0661
162.0660
168.0767
180.0766
m/z 180.0768
94.0398
96.0554
110.0712
138.0665
152.0817
162.0661
m/z 150.0662
122.0713
m/z 162.0662
94.0396
m/z

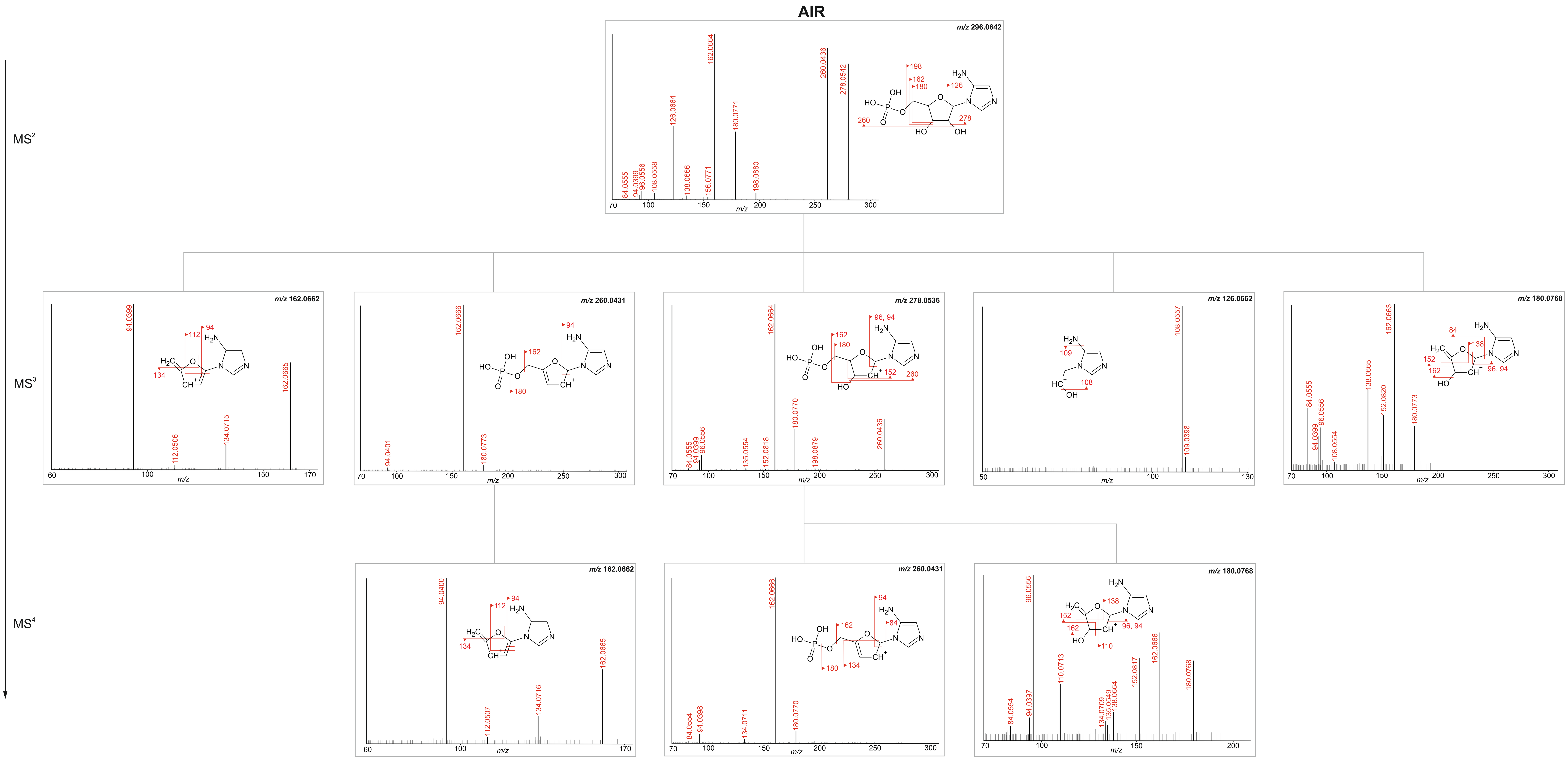

AIR
MS2
MS3
MS4
m/z 296.0642
84.0555
94.0399
96.0556
108.0558
126.0664
138.0666
156.0771
162.0664
180.0771
198.0880
260.0436
278.0542
m/z
m/z 162.0662
94.0399
112.0506
134.0715
162.0665
m/z 260.0431
94.0401
162.0666
180.0773
m/z 278.0536
84.0555
94.0399
96.0556
135.0554
152.0818
162.0664
180.0770
198.0879
260.0436
m/z 126.0662
108.0557
109.0398
m/z 180.0768
84.0555
94.0399
96.0556
108.0554
138.0665
152.0820
162.0663
180.0773
m/z 162.0662
94.0400
112.0507
134.0716
162.0665
m/z 260.0431
84.0554
94.0398
134.0711
162.0666
180.0770
m/z 180.0768
84.0554
94.0397
96.0556
110.0713
134.0709
135.0549
138.0664
152.0817
162.0666
180.0768

CAIr

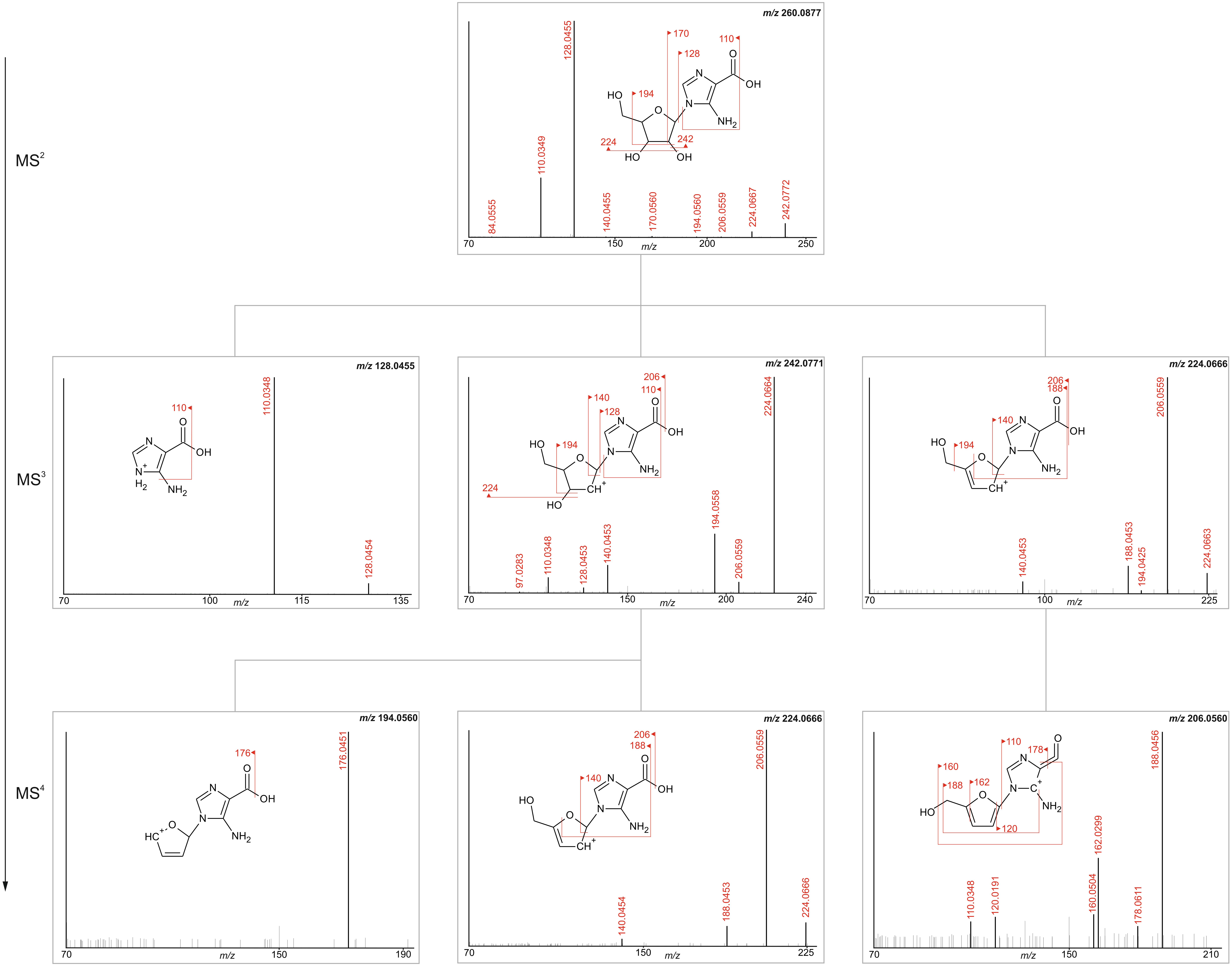
MS2
MS3
MS4
m/z 260.0877
84.0555
110.0349
128.0455
140.0455
170.0560
194.0560
206.0559
224.0667
242.0772
m/z 128.0455
110.0348
128.0454
m/z 242.0771
97.0283
110.0348
128.0453
140.0453
194.0558
206.0559
224.0664
m/z 224.0666
140.0453
188.0453
194.0425
206.0559
224.0663
m/z 194.0560
176.0451
m/z 224.0666
140.0454
188.0453
206.0559
224.0666
m/z 206.0560
110.0348
120.0191
160.0504
162.0299
178.0611
188.0456
m/z

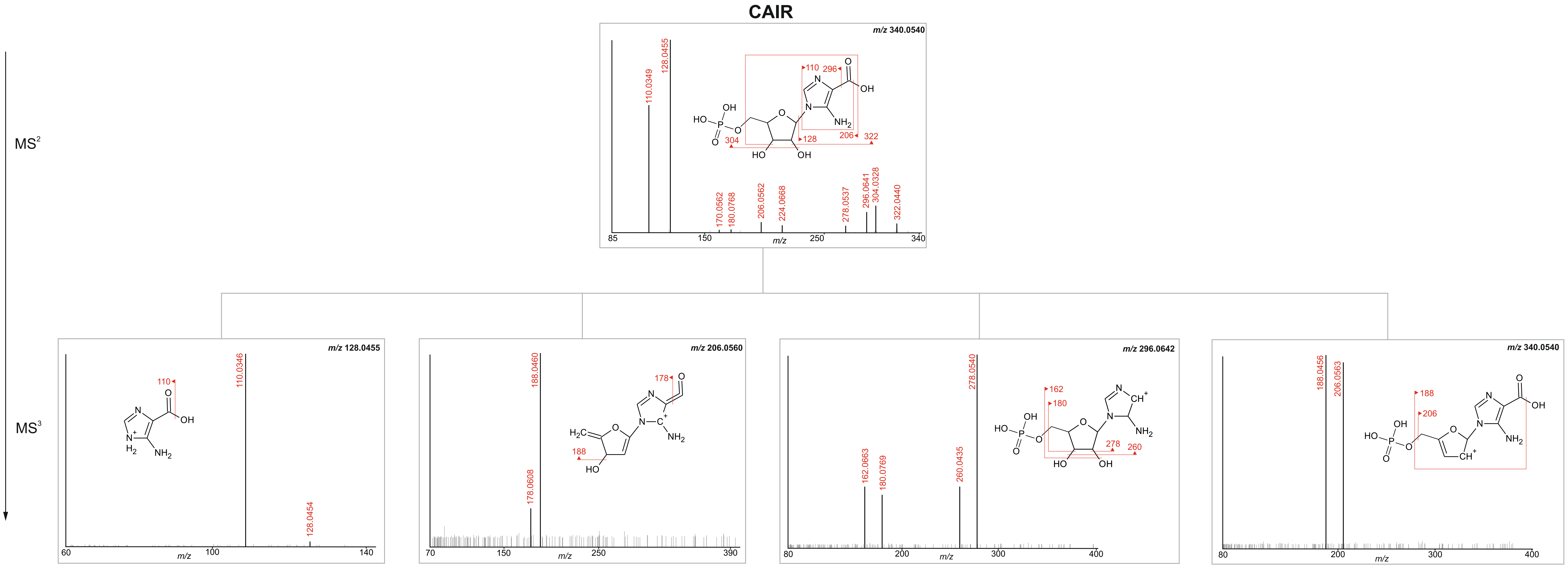
CAIR
MS2
MS3
m/z 340.0540
110.0349
128.0455
170.0562
180.0768
206.0562
224.0668
278.0537
296.0641
304.0328
322.0440
85
150
m/z
250
340
m/z 128.0455
110.0346
128.0454
60
100
140
m/z 206.0560
188.0460
178.0608
70
150
250
390
m/z 296.0642
162.0663
180.0769
260.0435
278.0540
80
200
300
400
m/z 340.0540
188.0456
206.0563
80
200
300
400

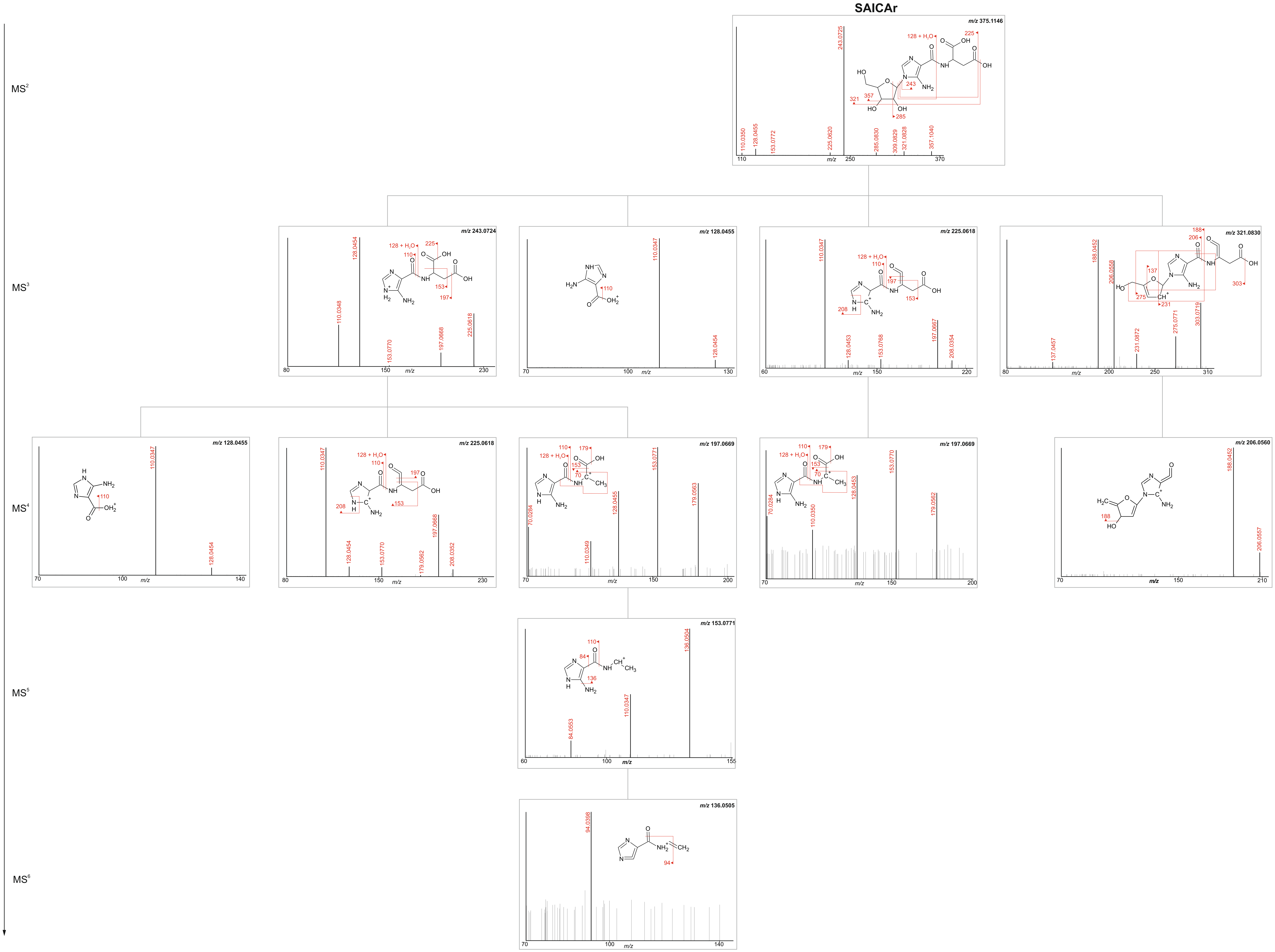
SAICAr
MS2
MS3
MS4
MS5
MS6
m/z 375.1146
m/z 243.0724
m/z 128.0455
m/z 225.0618
m/z 321.0830
m/z 128.0455
m/z 225.0618
m/z 197.0669
m/z 197.0669
m/z 206.0560
m/z 153.0771
m/z 136.0505
m/z

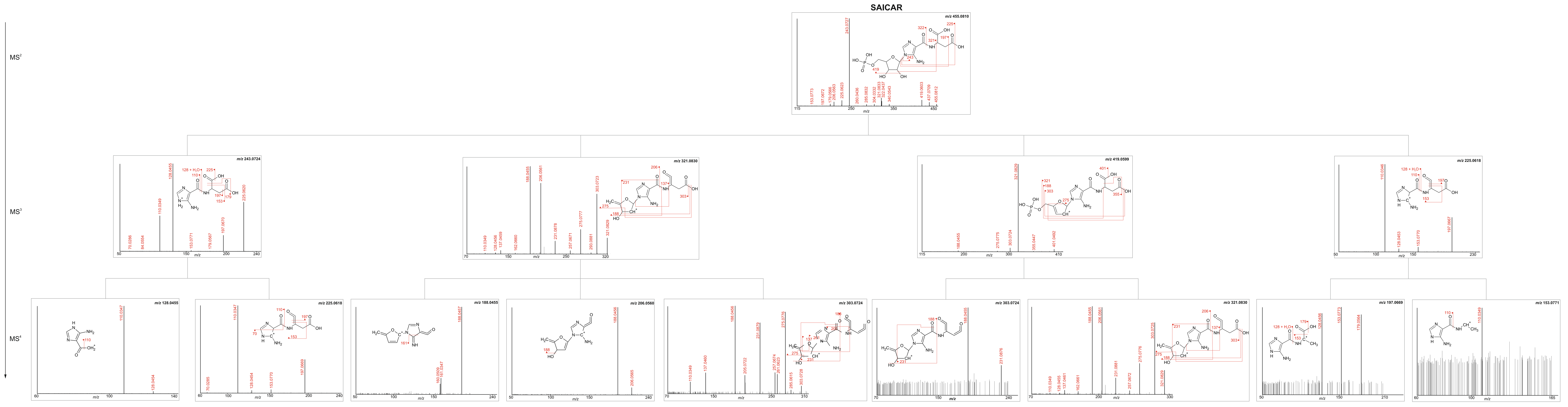

SAICAR
MS2
MS3
MS4
m/z 455.0810
m/z 243.0724
m/z 321.0830
m/z 419.0599
m/z 225.0618
m/z 128.0455
m/z 225.0618
m/z 188.0455
m/z 206.0560
m/z 303.0724
m/z 303.0724
m/z 321.0830
m/z 197.0669
m/z 153.0771

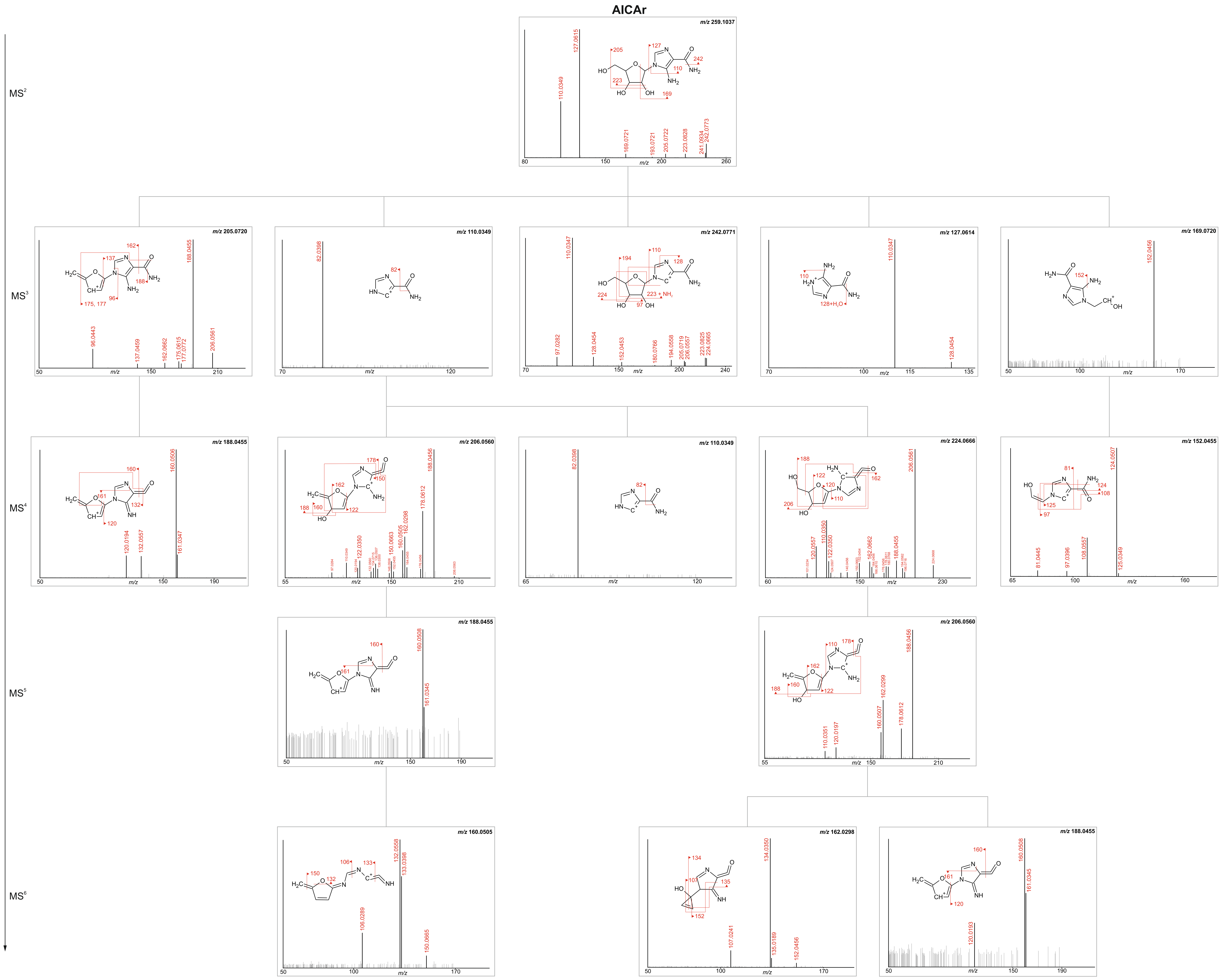

AICAr
MS2
MS3
MS4
MS5
MS6
m/z 259.1037
110.0349
127.0615
169.0721
193.0721
205.0722
223.0828
241.0934
242.0773
m/z 205.0720
96.0443
137.0459
162.0662
175.0615
177.0772
188.0455
206.0561
m/z 110.0349
82.0398
m/z 242.0771
97.0282
110.0347
128.0454
152.0453
180.0766
194.0558
205.0719
206.0557
223.0825
224.0665
m/z 127.0614
110.0347
128.0454
m/z 169.0720
152.0456
m/z 188.0455
120.0194
132.0557
160.0506
161.0347
m/z 206.0560
122.0350
150.0663
160.0505
162.0298
178.0612
188.0456
m/z 110.0349
82.0398
m/z 224.0666
120.0557
110.0350
122.0350
162.0662
188.0455
206.0561
m/z 152.0455
81.0445
97.0396
108.0557
124.0507
125.0349
m/z 188.0455
160.0508
161.0345
m/z 206.0560
110.0351
120.0197
160.0507
162.0299
178.0612
188.0456
m/z 160.0505
106.0289
132.0558
133.0398
150.0665
m/z 162.0298
107.0241
134.0350
135.0189
152.0456
m/z 188.0455
120.0193
160.0508
161.0345
m/z

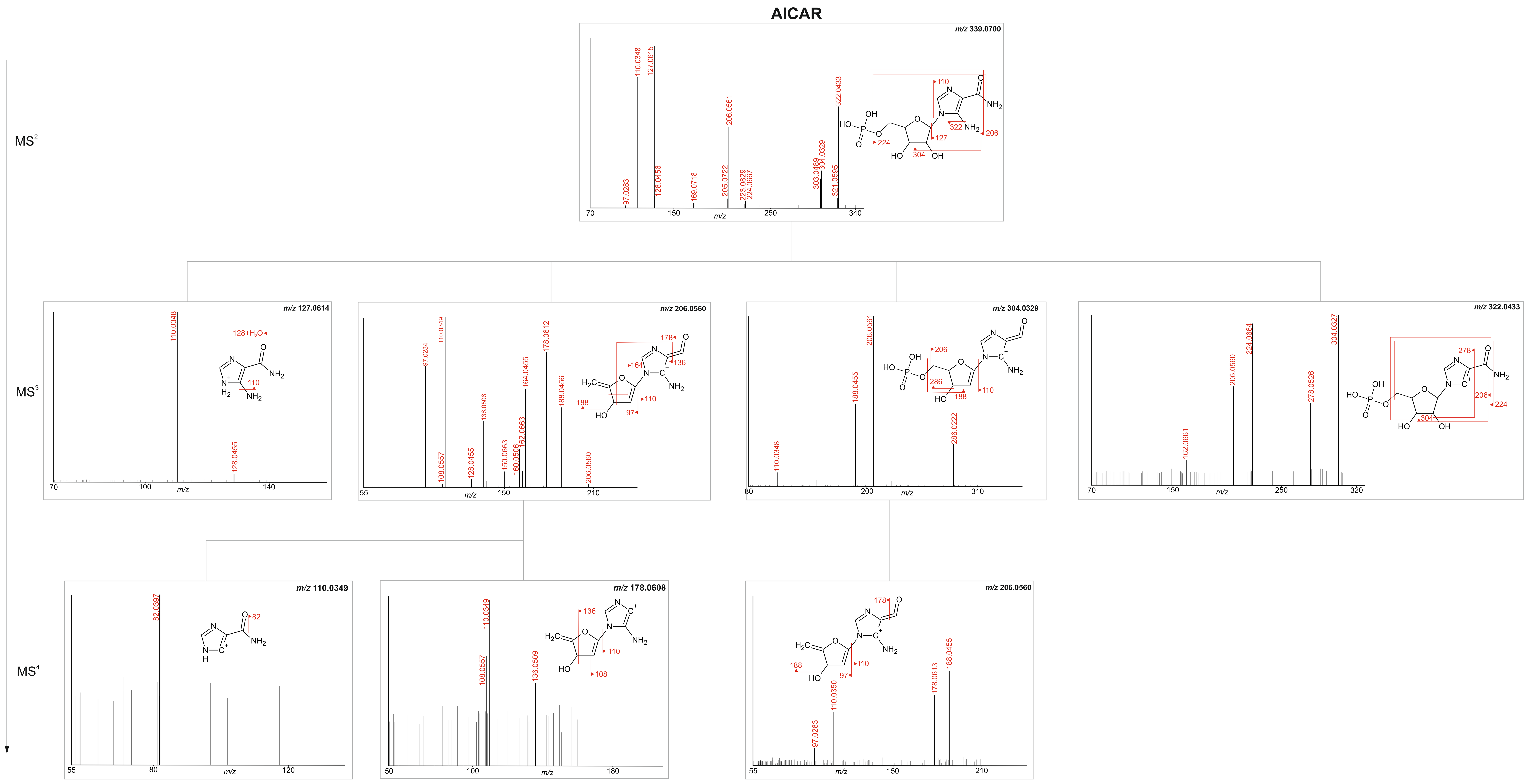

AICAR
MS2
MS3
MS4
m/z 339.0700
m/z 127.0614
m/z 206.0560
m/z 304.0329
m/z 322.0433
m/z 110.0349
m/z 178.0608
m/z 206.0560

FAICAr

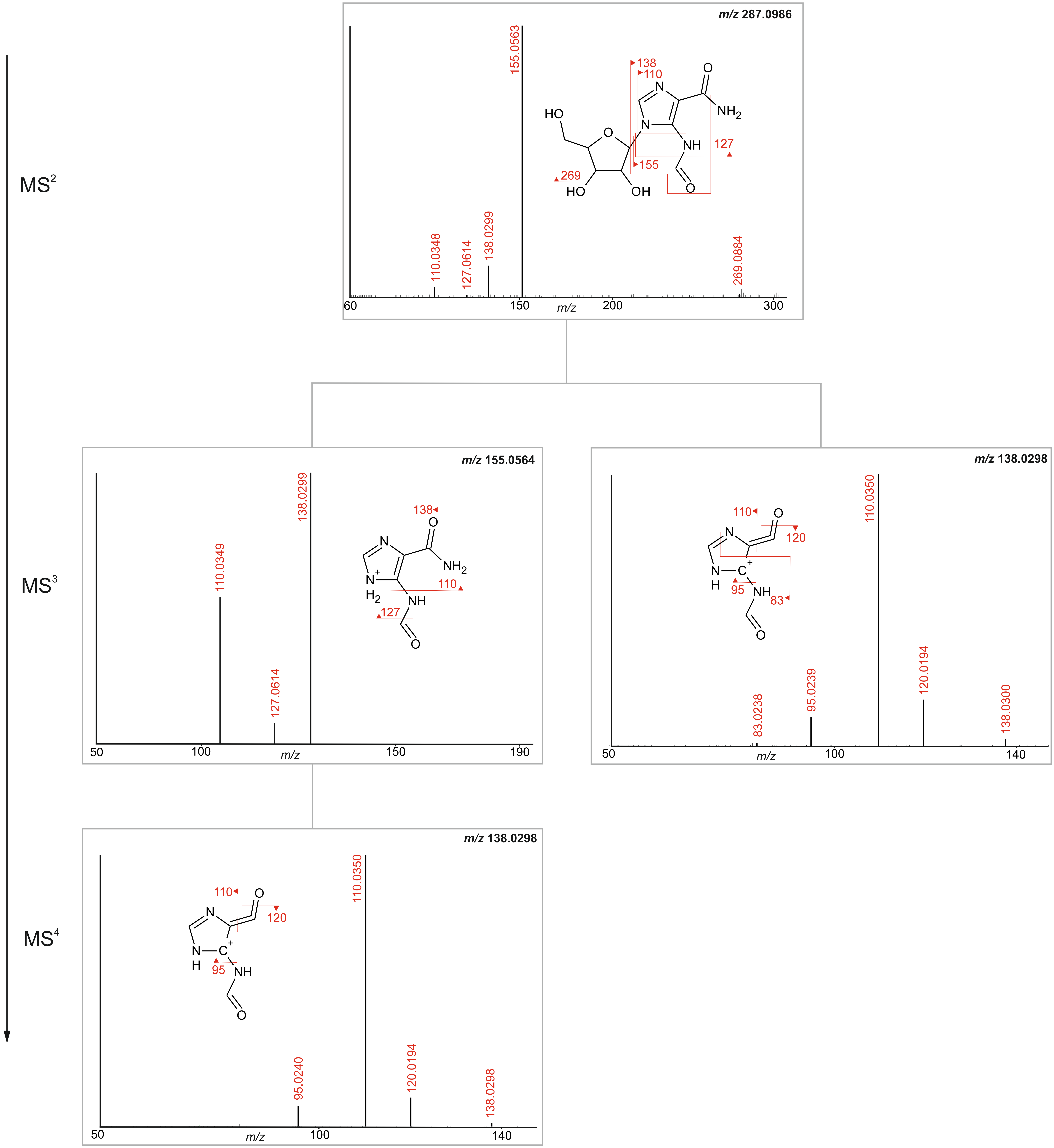
MS2
MS3
MS4
m/z 287.0986
155.0563
138.0299
127.0614
110.0348
269.0884
60
150
m/z
200
300
138
110
127
155
269
HO
O
N
NH2
NH
OH
m/z 155.0564
138.0299
110.0349
127.0614
50
100
150
190
m/z 138.0298
110.0350
120.0194
95.0239
83.0238
138.0300
120
95
83
140
m/z 138.0298
110.0350
95.0240
120.0194
138.0298

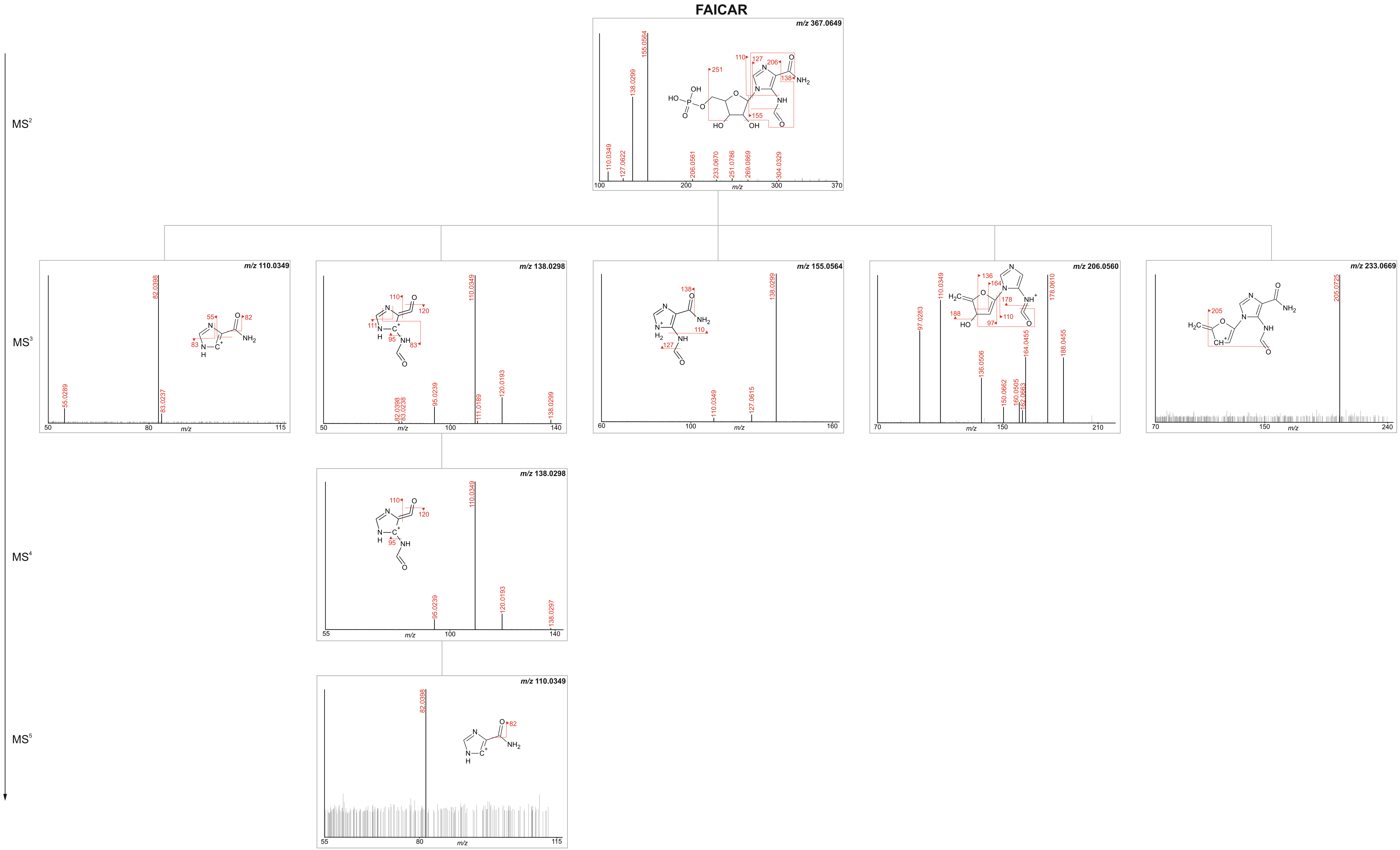
FAICAR
MS2
MS3
MS4
MS5
m/z 367.0649
m/z 110.0349
m/z 138.0298
m/z 155.0564
m/z 206.0560
m/z 233.0669
m/z 138.0298
m/z 110.0349
m/z

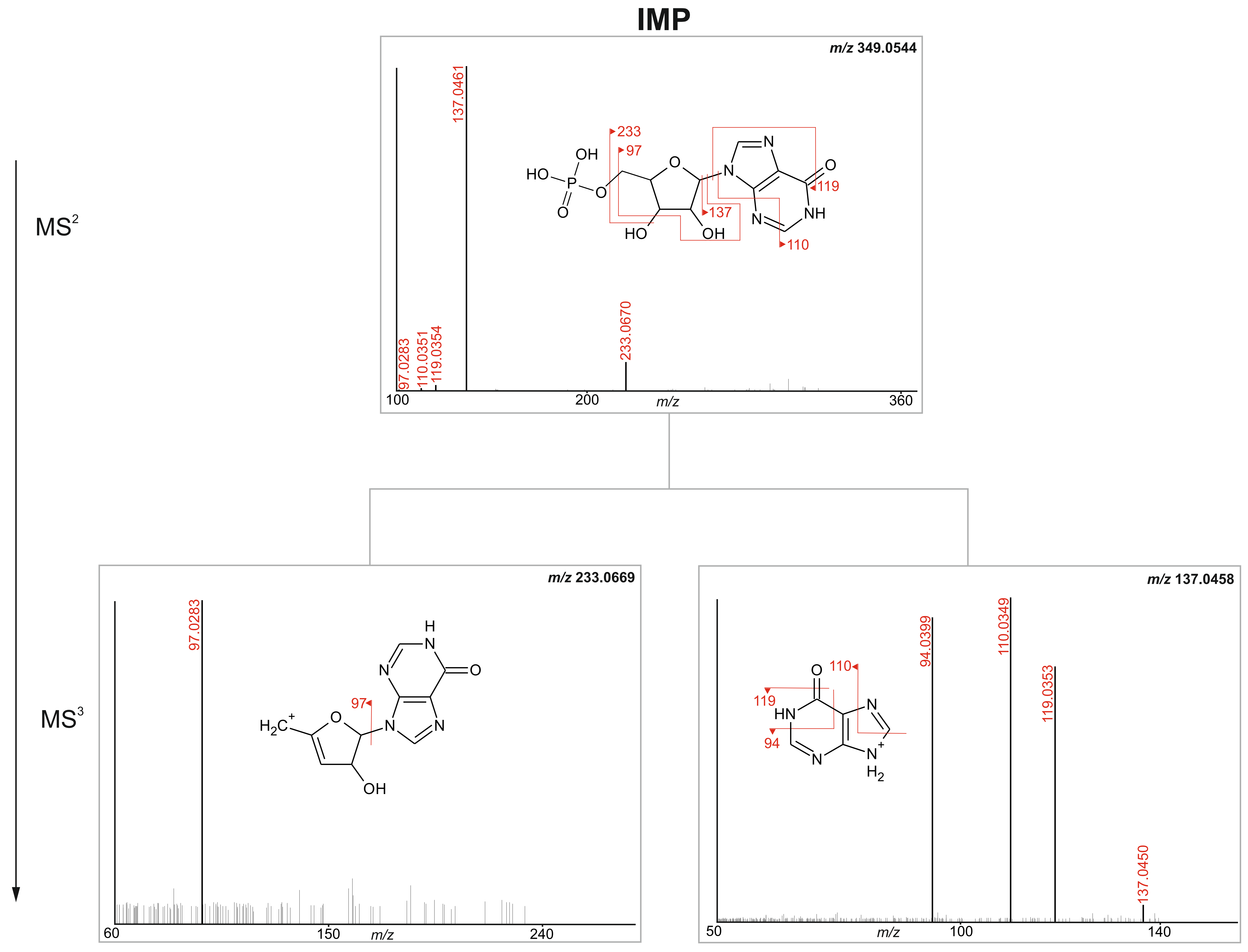
IMP
MS2
MS3
m/z 349.0544
137.0461
97.0283
110.0351
119.0354
233.0670
233
97
137
119
110
100
200
m/z
360
m/z 233.0669
97.0283
97
60
150
m/z
240
m/z 137.0458
94.0399
110.0349
119.0353
137.0450
110
119
94
50
m/z
100
140

SAdo

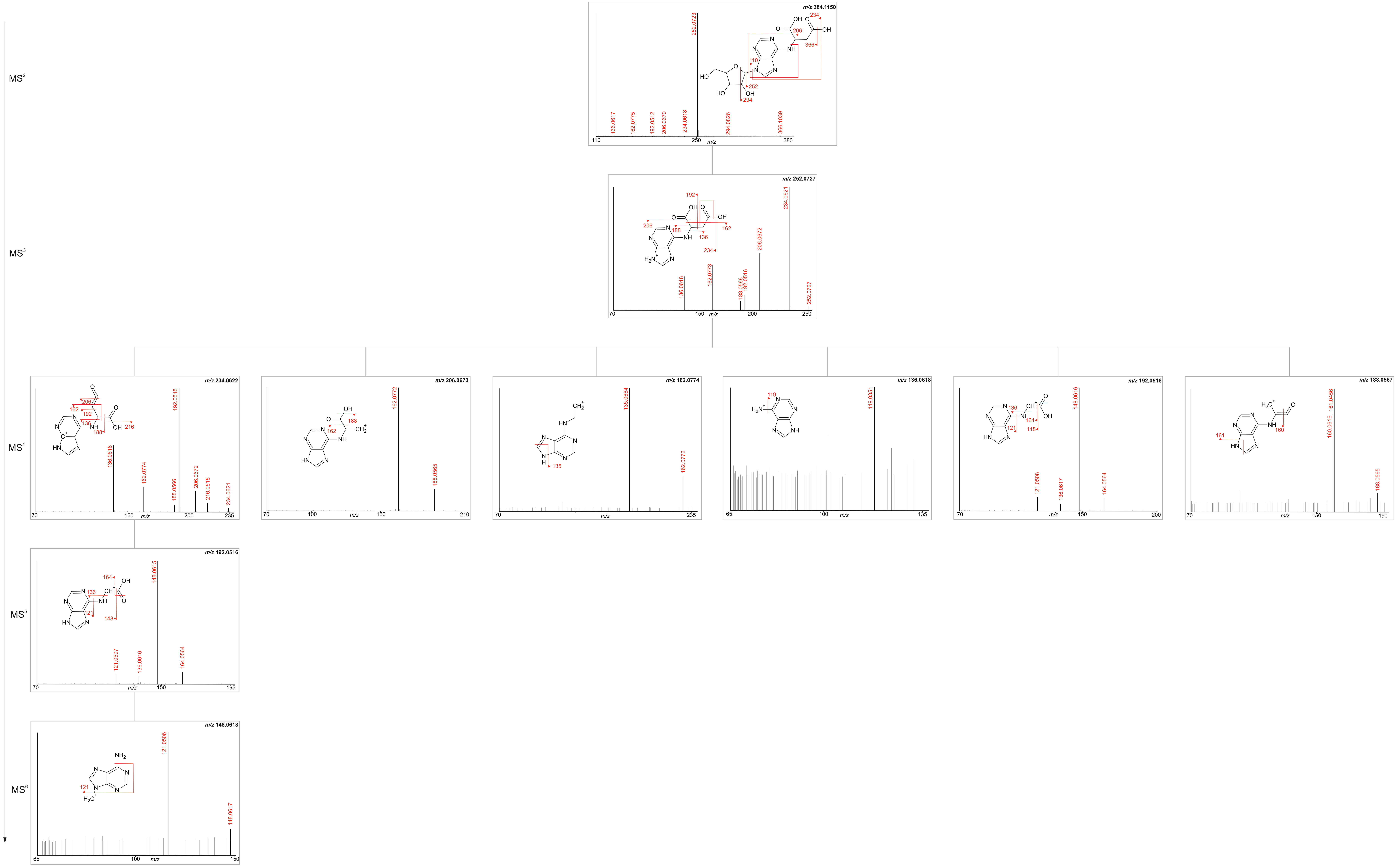

MS^2
MS^3
MS^4
MS^5
MS^6
m/z 384.1150
m/z 252.0727
m/z 234.0622
m/z 206.0673
m/z 162.0774
m/z 136.0618
m/z 192.0516
m/z 188.0567
m/z 192.0516
m/z 148.0618
m/z

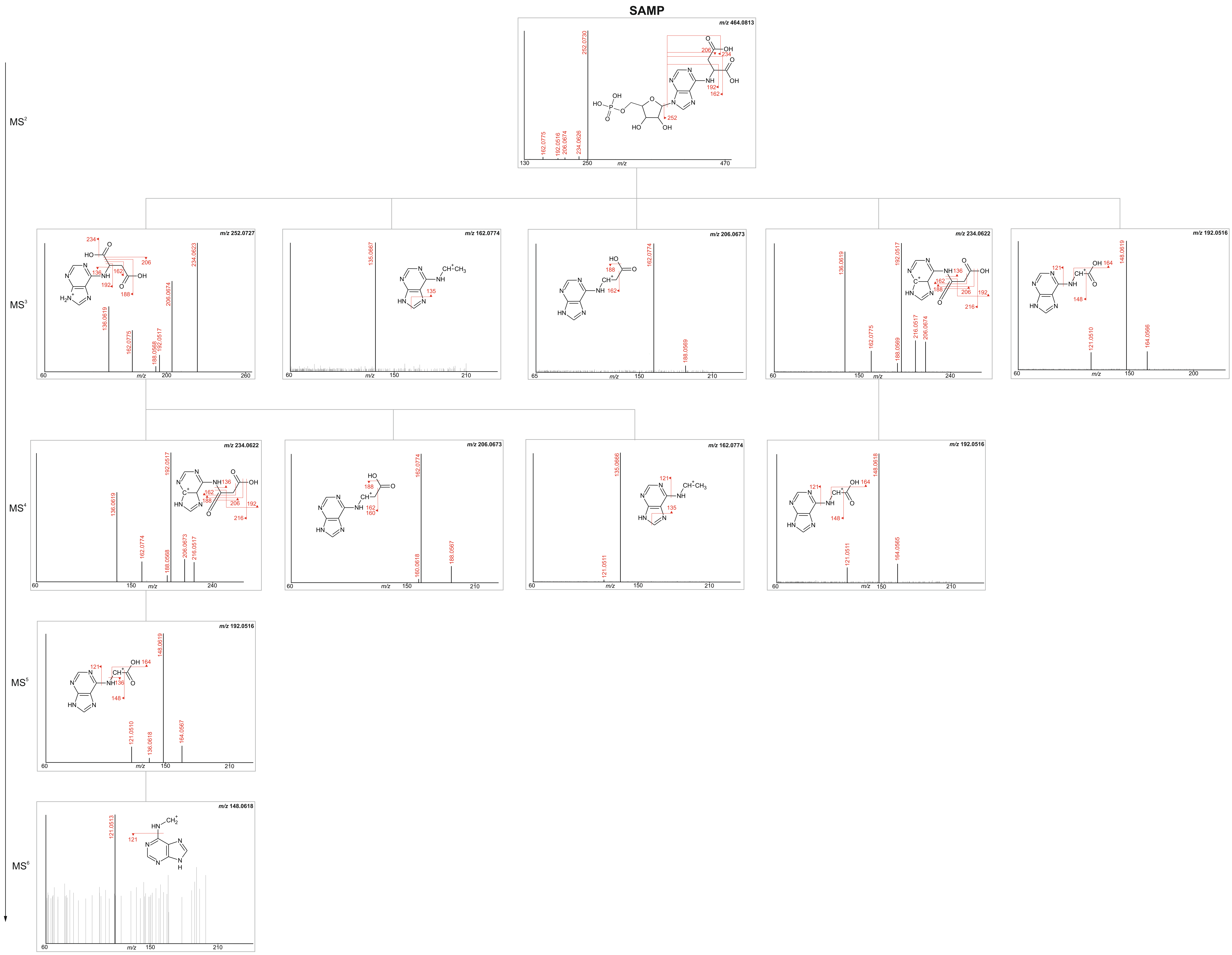

SAMP
MS2
MS3
MS4
MS5
MS6
m/z 464.0813
252.0730
162.0775
192.0516
206.0674
234.0626
m/z 252.0727
136.0619
162.0775
188.0568
192.0517
206.0674
234.0623
m/z 162.0774
135.0667
m/z 206.0673
162.0774
188.0569
m/z 234.0622
136.0619
162.0775
188.0569
192.0517
216.0517
206.0674
m/z 192.0516
121.0510
148.0619
164.0566
m/z 234.0622
136.0619
162.0774
188.0568
192.0517
206.0673
216.0517
m/z 206.0673
160.0618
162.0774
188.0567
m/z 162.0774
121.0511
135.0666
m/z 192.0516
121.0511
148.0618
164.0565
m/z 192.0516
121.0510
136.0618
148.0619
164.0567
m/z 148.0618
121.0513
m/z
